# Conservation and Divergence of *PEPC* Gene Family in Different Ploidy Bamboos

**DOI:** 10.3390/plants13172426

**Published:** 2024-08-30

**Authors:** Wenlong Cheng, Junlei Xu, Changhong Mu, Jutang Jiang, Zhanchao Cheng, Jian Gao

**Affiliations:** Key Laboratory of National Forestry and Grassland Administration, Beijing for Bamboo & Rattan Science and Technology, International Center for Bamboo and Rattan, State Forestry and Grassland Administration, Beijing 100102, China; chengwenlong@icbr.ac.cn (W.C.); xjl@icbr.ac.cn (J.X.); muchanghong@icbr.ac.cn (C.M.); jiangjutang@icbr.ac.cn (J.J.)

**Keywords:** Bambusoideae, phosphoenolpyruvate carboxylase (PEPC), evolution, gene expression pattern

## Abstract

Phosphoenolpyruvate carboxylase (PEPC), as a necessary enzyme for higher plants to participate in photosynthesis, plays a key role in photosynthetic carbon metabolism and the stress response. However, the molecular biology of the *PEPC* family of Bambusoideae has been poorly studied, and the function of its members in the growth and development of Bambusoideae is still unclear. Here, we identified a total of 62 PEPC family members in bamboo. All the *PEPC* genes in the bamboo subfamily were divided into twelve groups, each group typically containing significantly fewer *PEPC* members in *Olyra latifolia* than in *Phyllostachys edulis*, *Dendrocalamus latiflorus* and *Dendrocalamus brandisii*. The results of an intraspecific and interspecies collinearity analysis showed that fragment replication and whole genome replication were the main driving forces of bamboo *PEPC* members. Furthermore, the *Ka*/*Ks* values of collinear genes showed that bamboo *PEPC* experienced purification selection. In addition, the promoter region of *PEPC* genes contains cis-acting elements related to light response, plant hormone response and response to stress. An analysis of the expression levels of the *PEPC* family in different developmental stages and tissues of bamboo shoots has shown that *PhePEPC7*, *PhePEPC9* and *PhePEPC10* were highly expressed in the leaves of non-flowering plants and culms. Furthermore, *PhePEPC6* was significantly upregulated in leaves after GA treatment. Further research has shown that *PhePEPC6* was mainly localized in the cell membrane. This provides a solid bioinformatics foundation for further understanding the biological functions of the bamboo *PEPC* family.

## 1. Introduction

PEPC (Phosphoenolpyruvate carboxylase) is a cytoplasmic enzyme necessary for higher plants to participate in photosynthesis carbon metabolism [1]. At present, it has been found that PEPC mainly exists in some higher vascular tissue plants, blue-green algae and non-photosynthetic bacteria, while it has not been reported in animals and fungi [2,3]. In the process of green plants assimilating CO_2_ for photosynthesis, PEPC mainly relies on Mg^2+^ or Mn^2+^ and other cofactors to catalyze phosphoenolpyruvate (PEP) to decarboxylate and produce inorganic phosphate, oxaloacetic acid (OAA) and other derivatives [4]. Many studies have shown that PEPC has different functions in different plants and different tissues. C_4_ and Crassulacean acid metabolizers (CAM) use PEPC to fix atmospheric CO_2_ to produce photosynthetic organic products. In C_3_ plant leaves and some non-photosynthetic tissues, PEPC is mainly involved in the synthesis of intermediate metabolites in the citrate cycle (TCA), and these intermediate metabolites are finally used in various biosynthetic pathways [5,6,7,8]. In addition, PEPC is also involved in regulating seed and fruit development, stomatal opening of plant leaves and regulation of plants’ response to stress [9,10,11,12].

At present, research on PEPC in most higher plants has found that there are several small *ppc* gene families encoding PEPC [13]. Six *ppc* genes were found in rice [14] and sorghum [15], and three in sugarcane, maize and pineapple [16,17]. Four *ppc* genes were identified from the dicotyledonous plant *Arabidopsis thaliana* (*Atppc1–4*) [18]. Nine and ten *PEPC* genes were identified in cranberry, peanut [19] and soybean [20], and six *PEPC* genes were identified in the tropical plant durian [21]. PEPC mainly plays a role in fixing CO_2_ and participating in light and carbon assimilation in C_4_ and CAM plants [22]. Recent studies have shown that transferring the *PEPC* gene from C_4_ plant species into C_3_ plants can enhance their photosynthetic properties. Specifically, by overexpressing the *SiPEPC* gene in rice, it was observed that the transgenic plants exhibited higher light saturation points and lower CO_2_ compensation points compared to the control plants, ultimately leading to improved photosynthetic efficiency [23]. Transferring the maize *PEPC* gene into *Arabidopsis* revealed that the PEPC enzyme activity and photosynthetic rate of plants overexpressing the *ZmPEPC* gene were significantly increased in *Arabidopsis* [24]. In *Kalanchoe laxiflora*, silencing the *ppc1* affects stomata opening at night and CO_2_ fixation during the dark response, and also interferes with the stability of the circadian rhythm [25]. In addition to its significant role in the photosynthetic pathway, in C_3_ plants, PEPC can supplement the intermediate products required for the tricarboxylic acid cycle and catalyze the synthesis of OAA and subsequent synthesis of malic acid (Mal) and its derivatives to maintain the carbon skeleton and support synthetic metabolism [26].

The *PEPC* gene is widely present in various tissues of plants and may play different functions in different organs and tissues. Root-type PEPC may join in plant response to stress, and *Atppc3* is mainly expressed in the roots. *Atppc3* mutant lines are more sensitive to cadmium [27]. Sorghum Sbppc3 is mainly a protein that is specifically expressed in the roots. Its absence results in a lower yield per plant and delayed flowering. In addition, the silencing of *Sbppc3* negatively regulates sorghum’s response to salt stress [28]. Tomato *SlPEPC1* and *SlPEPC4* were significantly upregulated in the roots after six hours of drought treatment [29]. Rice overexpressing maize *ZmPEPC* showed higher drought resistance, with a higher dry weight and water use efficiency under drought conditions compared to the control plant [30]. The transcriptome abundance of soybean *GmPEPC3*, *GmPEPC6*, *GmPEPC8* and *GmPEPC9* significantly increased after salt stress treatment [18].

Bamboo is an excellent carbon sequestration tree because of its unique biological characteristics and excellent carbon sequestration ability. As an important forest carbon reservoir, the bamboo forest plays a crucial role in carbon sequestration and emission reduction of the forest ecosystem and regional carbon balance. Moso bamboo belongs to Gramineae and Bambusoideae and is one of the most widely distributed and important bamboo resources in China [31]. The rapid growth rate of bamboo indicates that it may have a strong assimilation ability, which is closely related to photosynthesis [32]. At the same time, bamboo has strong carbon sequestration ability, and the growth process of bamboo shoots is significantly correlated with carbon flux [33,34]. The study on PEPC activity in culms of bamboo shows that the PEPC activity gradually increases with the rapid growth of culms, and the photosynthetic carbon sequestration increases at this time, which is conducive to the rapid growth of bamboo shoots [35]. PEPC is a crucial enzyme that is heavily involved in the carbon assimilation process of photosynthesis in higher plants, and it serves as a vital regulatory function in the photosynthesis of plants. However, previous studies have only compared and analyzed the PEPC content in bamboo plants [36], and the molecular biology of the *PEPC* family has been poorly studied. The purpose of this study was to investigate the evolutionary relationship of the *PEPC* in bamboo plants from diploid to hexaploid, as well as its functions under abiotic stress, hormone response conditions and different growth and development stages. This study first identified the gene family members encoding *PEPC* from the genomes of diploid *O. latifolia*, tetraploid *P. edulis* and hexaploid *D. latiflorus* and *D. brandisii* and systematically analyzed their gene structure, conserved motifs, family evolution and gene spatiotemporal expression patterns. The identification of the *PEPC* gene family in bamboo plants helps us understand the differentiation of gene functions during bamboo polyploidization and also helps us design new strategies to respond to various environmental stresses.

## 2. Results

### 2.1. Identification and Physicochemical Properties of PEPC Members in Bamboos

A hidden Markov model was constructed to identify the *PEPC* gene family members. Only when the protein sequence contained the *PEPC* family motif (IPR021135), a lysine active site (IPR08129) and a histidine active site (IPR033129) was it identified as a member of the bamboo *PEPC* family [37]. After removing the PEPcase free conserved domain sequences, 10 *PEPC* members were identified in *P. edulis*. In addition, 7, 21 and 24 *PEPC* sequences were identified in *O. latifolia*, *D. latiflorus* and *D. brandisii*, respectively. It seems that during the process of bamboo chromosome replication, some *PEPC* genes were lost, resulting in a decrease in the number of *PEPCs*.

Naming was based on the position of the *PEPC* gene on the bamboo chromosomes. The protein sequence analysis of the *PEPC* gene family of Bambusoideae showed that the amino acid number of the PEPC protein ranged from 697 to 1485 aa. The molecular weight of the proteins ranged from 79.36 to 164.28 kD, and their theoretical isoelectric points were less than seven, which are negatively charged proteins. The instability coefficients were all greater than 40%, indicating that they were unstable proteins. Furthermore, the hydrophilic prediction analysis of the protein revealed that the fat solubility index was between 85.75 and 96.67, showing that it was a hydrophilic protein. In addition, the subcellular localization prediction analysis of PEPC protein in bamboo subfamily showed that PhePEPC proteins were located in the cytoplasm, while the OlaPEPC proteins were located in the chloroplasts. The extracellular proteins’ localizations were also found in the vacuole. Most of DlaPEPC proteins were located in the cytoplasm, but some members were located in the nucleus and plasma membrane. Except for DhPEPC2, DhPEPC13 and DhPEPC14 located in the chloroplast, all the other DhPEPC proteins were located in the cytoplasm of the *D. brandisii* (Table S1).

PEPC exists in various isoenzyme forms in plants, mainly divided into C_4_ type, C_3_ type and CAM type [2]. Due to the determination of PEPC protein isoenzymes by the 774 amino acid at the C-terminus of PEPC protein, C_4_ type PEPC protein is serine, C_3_ type is generally alanine and CAM plants are other amino acids [38]. In order to study the differences in the structural domains between the bamboo PEPC protein and C_4_ plant PEPC protein, we compared the amino acid sequences of bamboo and maize plant types PEPC. The results revealed that the amino acid sequences of bamboo PEPC were highly conserved. Moreover, the C-terminal 774 position of the protein peptide segment was alanine, suggesting that the bamboo PEPC protein belongs to the C_3_ type. All of them contain E/DR/KxxSIDAQ/LR phosphorylation sites near the N-terminal, and all contain amino acid sites related to PEPC enzyme activity (Figures S1 and S2).

### 2.2. Evolutionary Analysis of PEPCs Family

**Figure 1:**
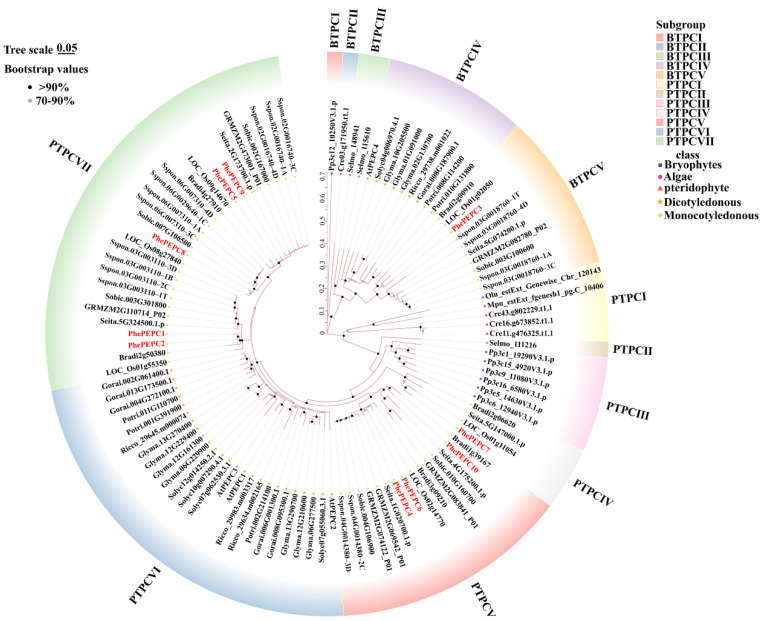
Phylogenetic tree of *PEPC* genes in 18 different species. A total of 108 PEPC proteins were identified from *P. edulis*, *A. thaliana*, *G. raimondii*, *P. trichocarpa*, *S. lycopersicum*, *G. max*, *R. communis*, *Z. mays*, *O. sativa*, *S. italica*, *S. bicolor*, *S. officinarum*, *B. distachyon*, *S. moellendorffii*, *P. patens*, *O. lucimarinus*, *C. reinhardtii* and *M. pusilla*. Branches with different colors represent different subgroups, red font represents the Moso bamboo *PEPC* gene, bootstrap values are represented by triangles of different sizes and solid and dashed lines represent evolutionary distances.

In order to study the evolutionary relationship of the *PEPC* gene family, MEGA11.0 was applied to perform a phylogenetic analysis of 108 PEPC protein sequences in algae, bryophytes, ferns, monocotyledonous and dicotyledonous plants and bamboo by using the neighbor-joining method. The phylogenetic analysis revealed that the proteins of PEPC from 18 different species can be divided into BTPC and PTPC based on their level of similarity. The BTPC subfamily mainly consists of 23 members and is further divided into five branches, BTPCI to BTPCV. Except PhePEPC3 from the BTPC subfamily, other PEPC proteins belong to the PTPC subfamily. They were mainly concentrated in the three subfamilies of PTPCIV, PTPCV and PTPCVII. A homologous relationship analysis showed that the bamboo was closely related to monocotyledonous plants, such as rice, maize and *B. distachyon*, and the sequence consistency among members of the same subfamily was high, which indicates they may have evolved from the same gene. In addition, a phylogenetic analysis revealed hierarchical topological relationships between monocotyledonous and dicotyledonous plants, indicating that *PEPC* gene differentiation occurs before monocotyledonous and dicotyledonous plant differentiation (Figure 1).

**Figure 2:**
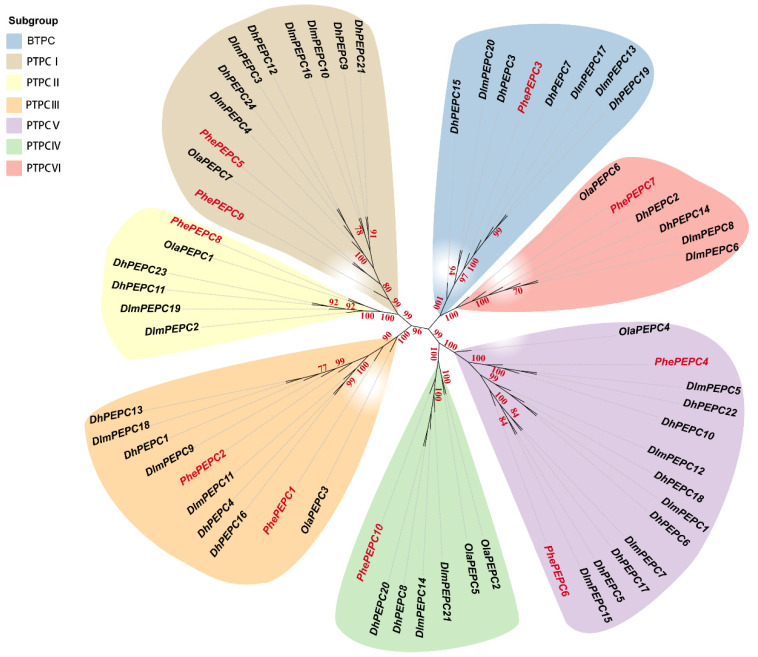
Phylogenetic tree of *PEPC* gene in *P. edulis*, *O. latifolia*, *D. latiflorus* and *D. brandisii*. Seven *OlaPEPC genes*, 10 *PhePEPC genes*, 21 *DlmPEPC genes* and 24 *DhPEPC genes* are clustered into seven subgroups. Branches with different colors represent different subgroups, and red font represents the Moso bamboo *PEPC* gene. The red numbers on the branches represent the bootstrap values with a credibility greater than 70.

Bamboo plants have multiple ploidies, and the evolution of the bamboo subfamily was driven by polyploidy. The copy number of the *PEPC* gene in polyploid woody bamboo was significantly higher than that in diploid herbaceous bamboo (Table S1). The phylogenetic tree of Bambusoideae was constructed using 62 PEPC protein sequences. It was found that the PEPCs of Bambusoideae were also divided into BTPC and PTPC subfamilies. Among them, OlaPEPC exists only in the PTPC subfamily, and PEPC presents hierarchical topological relationships in the PTPC subfamily (Figure 2).

### 2.3. Analysis of Gene Structure, Conservative Motif and Promoter Cis-Regulatory Element

**Figure 3:**
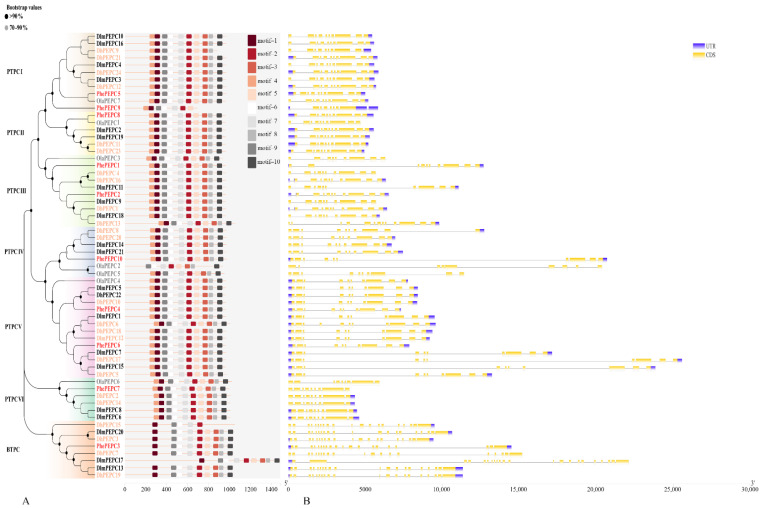
Phylogenetic relationships, conserved motifs and gene structures of *PEPC* genes in *P. edulis*, *O. latifolia*, *D. latiflorus* and *D. brandisii*. (**A**) The phylogenetic and conserved motifs of *PEPC* genes in *P. edulis*, *O. latifolia*, *D. latiflorus* and *D. brandisii*. Different colored blocks represent different conserved motifs identified. The gray, red, black, and orange fonts represent the *PEPC* gene members of *P. edulis*, *O. latifolia*, *D. latiflorus* and *D. brandisii*, respectively. (**B**) The *PEPC* gene structure of the *P. edulis*, *O. latifolia* and *D. latiflorus* and *D. brandisii*. The yellow box represents exon, the gray line connecting exon represents intron, and the blue box represents the untranslated region.

A gene structure analysis showed that there were differences in the length of coding sequences of 62 *PEPC* genes, which ranged from 2094 to 4458 bp. In addition, the BTPC subfamily and PTPC subfamily have significant differences in gene structure, with eight BTPC subfamily members (*PhePEPC3*, *DlaPEPC13*, *DlaPEPC17*, *DlaPEPC20*, *DhPEPC3*, *DhPEPC7*, *DhPEPC15*, *DhPEPC19*) containing 20 exons and 19 introns. In addition to the PTPCVI subfamily (*OlaPEPC6*, *PhePEPC7*, *DlaPEPC6*, *DlaPEPC8*, *DhPEPC2*, *DhPEPC14*), which contains only nine exons and eight introns, *PhePEPC9* contains seven exons and seven introns; the other 47 *PEPC* genes contain 10 exons and 9 introns (Figure 3). The difference in the number of introns and exons of *PhePEPC9* reveals its potential functional differentiation.

By using MEME software (V5.5.7) to predict the PEPC protein sequence of Moso bamboo, a total of 10 conserved motifs were obtained. Except for PhePEPC9, which lacks motifs 2 and 8, and OlaPEPC2, which lacks motifs 3 and 6, all the PTPC members contain 10 conserved motifs. Except for DhPEPC15, which only contains 5 motifs, all the other BTPC subfamily members contain nine motifs, lacking one motif 4. In addition, subfamilies within the same branch have relatively similar positions of conserved motifs in their PEPC proteins (Figure 3).

**Figure 4:**
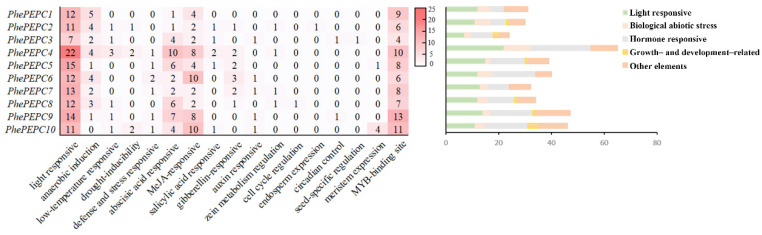
Analysis of the functional components of the *PEPC* promoter region in Moso bamboo. The rounded rectangular squares with different colors represent different cis-regulatory elements in the promoter area.

Furthermore, through the analysis of the cis acting elements of the *PEPC* family promoter in Moso bamboo, the results showed that all the *PEPC* family members contained light and anaerobic induced response elements. In addition, the *PEPC* gene family was also involved in plant hormone response, and all the genes contained abscisic acid (ABA) and methyl jasmonate (MeJA) response elements. All the genes except *PhePEPC1*, *PhePEPC9* and *PhePEPC10* contained gibberellin (GA) response elements; *PhePEPC2*, *PhePEPC4*, *PhePEPC5* and *PhePEPC10* promoter regions contained salicylic acid (SA) response elements; *PhePEPC2*, *PhePEPC4*, *PhePEPC7* and *PhePEPC8* contained zein metabolic elements; and *PhePEPC6*, *PhePEPC7*, *PhePEPC9* and *PhePEPC10* contained auxin response elements. The *PhePEPC2* promoter region also contained specific endosperm expression elements, suggesting that *PhePEPC2* may be involved in regulating the formation of a bamboo embryo. Only *PhePEPC8* has cell cycle regulation elements, while *PhePEPC5* and *PhePEPC10* have meristem expression elements. *PhePEPC5* and *PhePEPC9* contained circadian rhythm control elements. *PhePEPC2*, *PhePEPC4*, *PhePEPC8*, *PhePEPC9* and *PhePEPC10* contained response elements in response to low temperature and drought, and *PhePEPC4*, *PhePEPC5*, *PhePEPC6*, *PhePEPC7*, *PhePEPC9* and *PhePEPC10* all contained defense and stress response elements, indicating that most of the *PEPC* family of Moso bamboo respond to stress injury (Figure 4).

### 2.4. Collinearity Analysis and Evolutionary Pressure of PEPC in Bamboo

**Figure 5:**
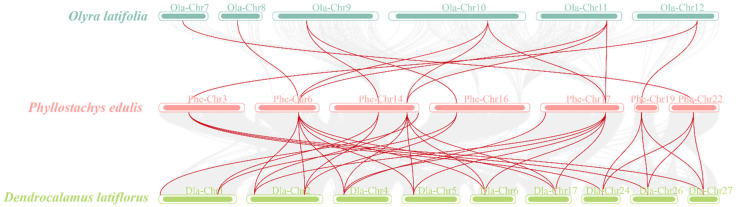
Colinear analysis of *PEPC* genes in *P. edulis*, *O. latifolia* and *D. latiflorus*. The squares with different colors represent chromosomes of different bamboo species, and the red lines represent homeotic gene pairs with collinear fragments.

**Figure 6:**
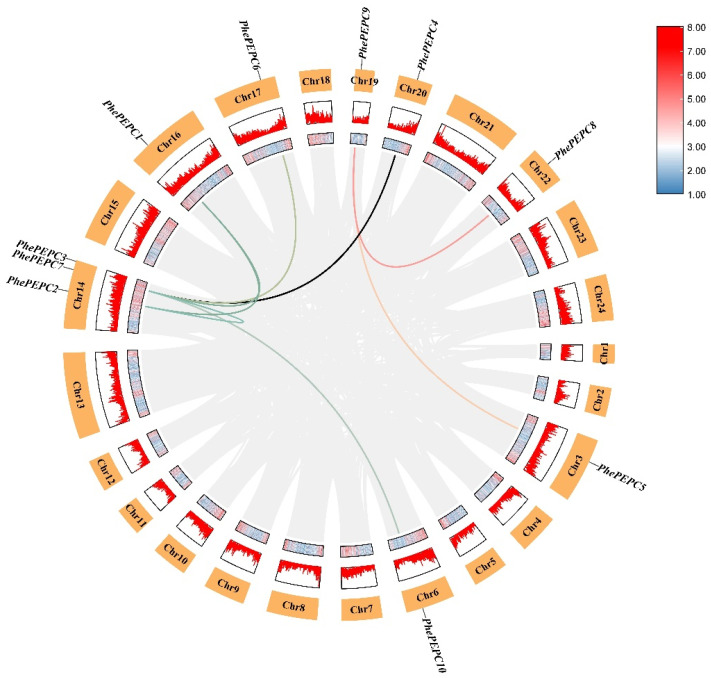
Collinearity analysis of the *PEPC* family in *P. edulis*. Different colored lines represent collinear pairs of *PhePEPC*.

The number of *PEPC* members varies greatly in diploid and hexaploid bamboo, and we hypothesized that the *PEPC* gene family is the result of gene amplification caused by chromosome doubling. To validate this hypothesis, we used MCScanX software (V1.3.1) to analyze the genomic collinearity among diploid bamboo, tetraploid bamboo and hexaploid bamboo. The results indicated that homologous genes of the *PEPC* gene appeared in collinear segments of different genomes. Furthermore, five *O. latifolia PEPC* genes share common ancestors with *P. edulis* and *D. latiflorus* (Figure 5). These results offer compelling evidence that *PEPC* primarily achieves gene amplification through whole genome replication (WGD) or segmental duplications. In addition, a collinearity analysis of the *PhePEPC* genes in bamboo revealed eight pairs of homologous *PhePEPCs*, indicating that these eight pairs were formed through fragment replication (Figure 6).

To explore the evolutionary pressure on *PEPC* genes across different bamboo species, an analysis of evolutionary pressure was carried out on orthologous and paralogous homologous *PEPC* gene pairs in *O. latifolia*, *P. edulis* and *D. latiflorus*. The results showed that both orthologous and paralogous *PEPC* homeotic gene pairs exhibited strong purification and selection (*Ka/Ks* < 0.5). The *Ks* value can be used to indicate the time when a gene replication event occurred. In diploid *O. latifolia*, the *Ks* of the accessory homeotic gene are higher than that of most accessory homeotic gene pairs in polyploid woody bamboos (Table S2), indicating that the formation of accessory homeotic gene copies of herbaceous bamboos may be caused by large segments of DNA replication before bamboo species differentiation.

### 2.5. Expression Patterns of the PEPC Family in P. edulis

**Figure 7:**
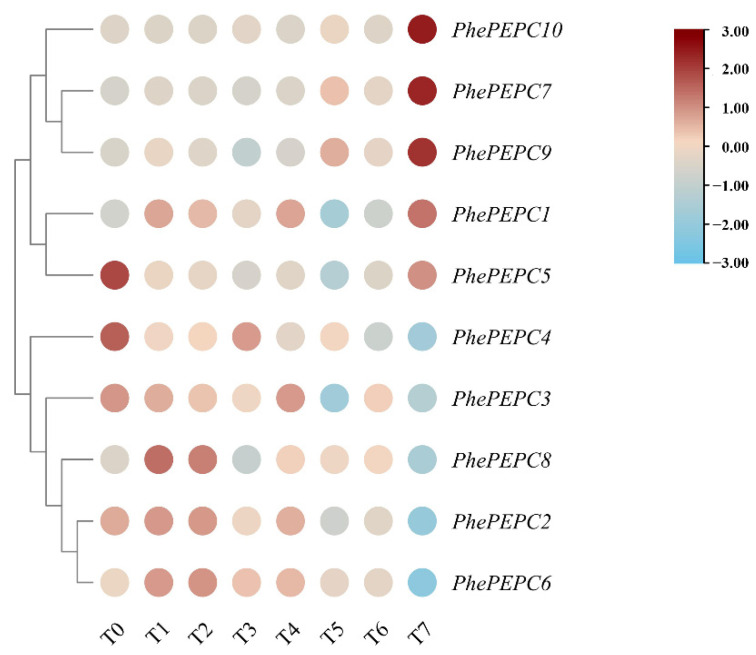
Expression level of *PEPC* gene in different stages of Moso bamboo shoot development. T0. winter bamboo shoots, T1. 50 cm, T2. 100 cm, T3. 300 cm, T4. 600 cm, T5. 900 cm, T6. 1200 cm, T7. culms of fully unfolded leaves.

**Figure 8:**
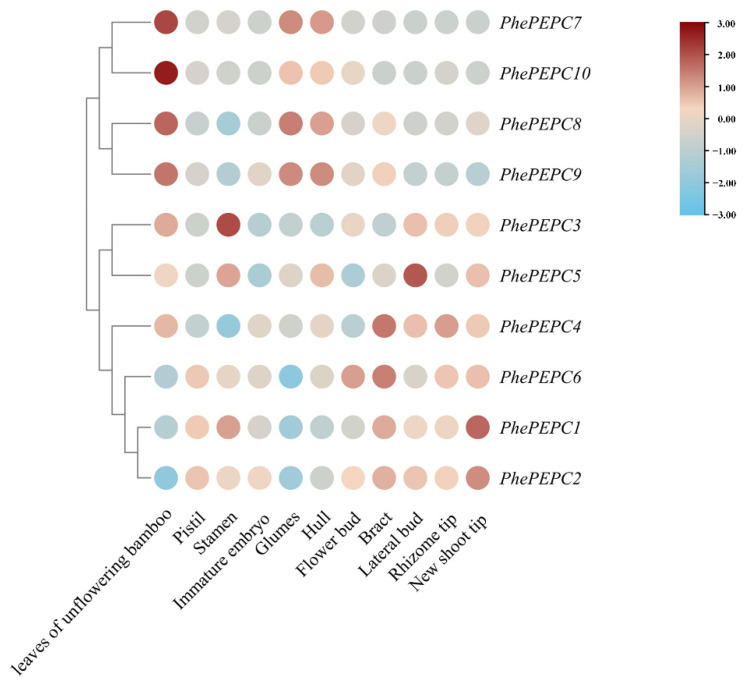
The relative expression of *PEPC* family in different tissues of Moso bamboo.

To explore the role of *PEPC* genes in the growth and development of bamboo shoots, we assessed the expression of *PhePEPCs* in different stages of shoot growth (winter shoots, 50 cm, 100 cm, 300 cm, 600 cm, 900 cm and 1200 cm shoot tips and culms of fully unfolded leaves) and flower organs (flower buds, bracts, glumes, lemmas, pistils, stamens, young embryos and leaves of a non-flowering bamboo plant) [39]. An analysis of the transcriptome data revealed that *PhePEPC1* was predominantly expressed in the culms of Moso bamboo. *PhePEPC7*, *9* and *10* were highly expressed in the culms and leaves of a non-flowering bamboo plant, while *PhePEPC4* and *PhePEPC5* were mainly expressed in winter bamboo shoots. The expression patterns of *PhePEPC2*, *6* and *8* were similar, mainly playing a major regulatory role in the early stage of shoot growth and development (Figure 7). In addition, the expression patterns of *PhePEPC4* and *PhePEPC6* in the flower organs were similar. Except for low expression in the leaves of a non-flowering bamboo plant and glumes, the expression levels were higher in immature embryos, flower buds and bracts, while *PhePEPC2* was significantly higher in the flower buds and bracts than in other tissues (Figure 8).

**Figure 9:**
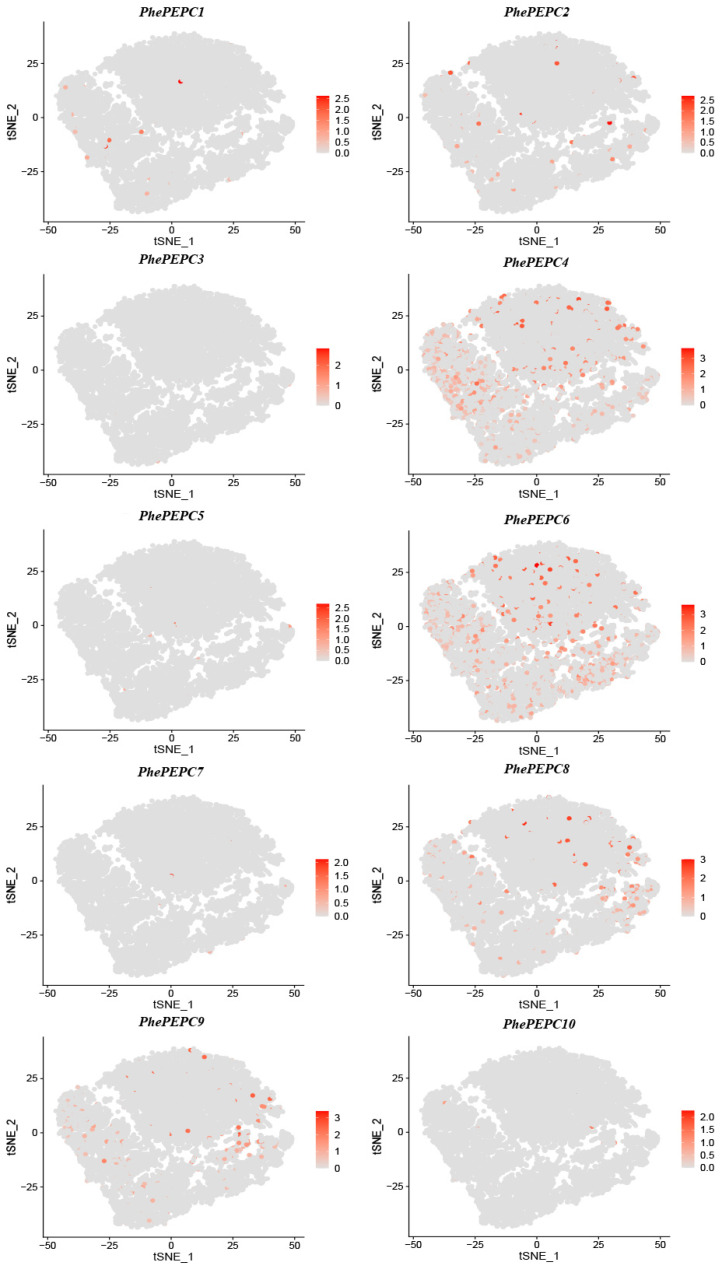
Visualization of *PhePEPCs* in scRNA-seq data of Moso bamboo basal roots. The depth of the color represents the expression of the genes.

The *PEPC* gene exists in various tissues of plants. In order to explore the specific expression site of the *PhePEPC* gene in Moso bamboo basal roots, in this study, we used single-cell transcriptome sequencing data from previous measurements of bamboo basal roots to study the *PEPC* gene family of bamboo [40]. We found that *PhePEPC2* was mainly expressed in the basic tissue group cluster, while *PhePEPC5* was specifically expressed in the root cap. In addition, *PhePEPC6*, *PhePEPC8* and *PhePEPC9* genes were all expressed in the basic tissues and root cap clusters (Figure 9 and Figure S2).

### 2.6. PhePEPCs Responds to the Exogenous GA Treatments

**Figure 10:**
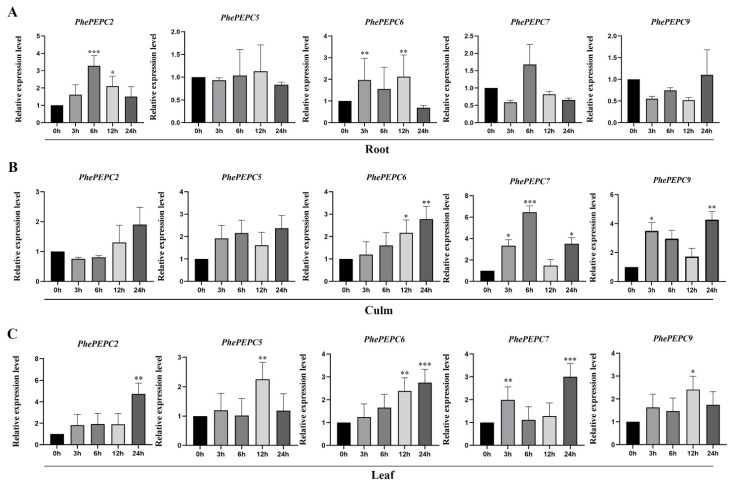
qRT-PCR analysis of *PhePEPC* genes in Moso bamboo after exogenous GA treatment. (**A**–**C**) represent expression patterns of 5 *PEPC* genes in the root, culm and leaf after GA treatment. *PheTIP41* was used as a reference gene in Moso bamboo. The error bars represent the standard deviation of the means of three biological replicates of each sample (*n* = 3). * Analysis of the difference in expression levels between representative and control (0.01 ≤ *p* ≤ 0.05 *, *p* < 0.01 **, *p* < 0.001 ***).

Due to the presence of GA responsive elements in the promoter region, these four genes were selected for exogenous GA treatment in this study. Under exogenous GA treatment, their roots, culms and leaves showed obvious oscillatory changes (Figure 10). After 100 μM GA_3_ treatment of Moso bamboo seedlings, the expression of *PhePEPC2* transcripts in the culms showed a trend of first increasing and then decreasing and reached its peak at six hours of treatment. *PhePEPC5* was not significantly expressed in the roots and culms after GA treatment but significantly increased in the leaves after 12 h of GA treatment. In addition, as the time point of GA treatment increased, the expression level of *PhePEPC6* in the leaf transcripts gradually increased, suggesting that it responds to GA treatment and participates in leaf growth and development. In the later stage of GA treatment, the expression of *PhePEPC7* significantly increased in the culms and leaves compared to the control.

### 2.7. Subcellular Localizations of PhePEPC Proteins

**Figure 11:**
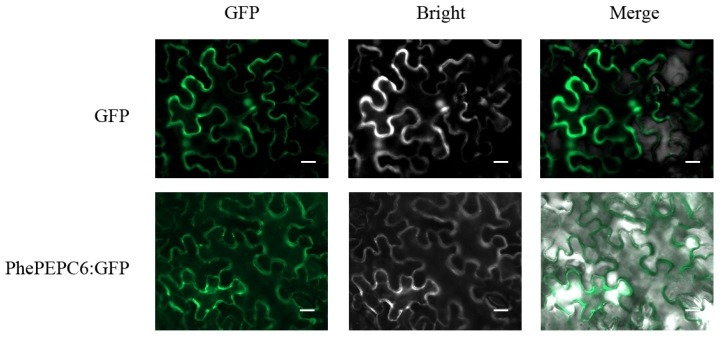
Subcellular localization of PhePEPC6. Bars = 10 μm.

To confirm the subcellular localization of the PhePEPC protein predicted by PlantmPLoc, we selected C_3_ photo zyme PhePEPC6 for a tobacco subcellular localization experiment. First, we cloned the full-length CDS sequence of *PhePEPC6* gene, then constructed it into a *PCAMBIA23300-35S-eGFP* vector and injected it into tobacco leaves through Agrobacterium infection. The results showed that the PhePEPC6 protein was mainly localized to the cell membrane compared with the GFP unloaded control (Figure 11).

## 3. Discussion

Phosphoenolpyruvate carboxylase plays a major role in fixing CO_2_ and maintaining photosynthetic carbon assimilation in C_4_- and CAM-metabolizing plants. In C_3_ plants, PEPC is mainly involved in tricarboxylic acid cycle (TCA) to compensate for oxaloacetic acid OAA’s function [5,6,7,8]. In addition, PEPC plays a wide range of roles in maintaining carbon–nitrogen interactions [37,41], seed formation and germination [42] and fruit ripening [26]. It has been reported that PEPC is also involved in plants’ response to abiotic stresses, such as drought [19,43,44,45], cold stress and heat stress [19,46,47].

Research of the molecular biology of bamboo *PEPC* genes remains scarce. In this study, we identified 10 *PEPC* members within the Moso bamboo genome, a number significantly greater than that found in Arabidopsis (4) and rice (6). The genome of bamboo was 1.91G [48], which was much larger than that of Arabidopsis and rice. The occurrence of genome replication events in bamboo may lead to an increase in the number of *PEPC* family members. In order to study the evolutionary relationship of the *PEPC* gene among different species of Bambusoideae, we also identified the members of the *PEPC* gene family of diploid *O. latifolia* and hexaploid *D. latiflorus* and *D. brandisii*. Seven *PEPC* members were identified in *O. latifolia*, and 21 *PEPC* genes were identified in *D. latiflorus*. Furthermore, 24 *PEPC* genes were identified in *D. brandisii*. We speculated that this might be due to the amplification of *PEPC* genes in *D. latiflorus* and *D. brandisii* during its evolution due to chromosome doubling. In addition, a thorough examination of collinearity events and *Ka*/*Ks* values revealed that whole genome replication and fragment replication were the primary mechanisms of amplification in the *PEPC* family, which is subject to continuous purification selection throughout evolution. Furthermore, a collinearity analysis between *P. edulis*, *O. latifolia* and *D. latiflorus* indicated that members of the *PEPC* gene family exhibit a certain degree of functional conservation during the evolution of the bamboo subfamily, highlighting the possibility of similar biological functions among different species.

A phylogenetic analysis of *PEPC* in different species revealed that the *PEPC* was mainly divided into two subfamilies: PTPC (plant type PEPC) and BTPC (bacterial type PEPC) [14]. The PTPC gene encodes a peptide of 100–110 kDa, with a conserved serine phosphorylation site at the N-terminus and a conserved QNTG tetrapeptide at the C-terminus [4]. The BTPC gene encodes a 116–118 kDa peptide with a tetrapeptide of RNTG at the C-terminus [49]. A phylogenetic analysis of bamboo PEPC proteins showed that except for PhePEPC3 belonging to the BTPC subfamily, all the other bamboo PEPC proteins belong to the PTPC subfamily and are mainly concentrated in the three subfamilies of PTPC IV, PTPC V and PTPC VII. In addition, the phylogenetic analysis revealed that the BTPC type was not present in *O. latifolia*. This might be due to incomplete genome assembly in the diploid herbaceous bamboo. Through the analysis of the phylogenetic tree of *PEPC* in different species, it can be concluded that almost all members of *PEPC* in Moso bamboo are clustered in monocotyledonous plant subgroups, while their evolutionary relationship with dicotyledonous plants, such as Arabidopsis, is relatively distant, indicating that *PEPC* genes may have existed before monocotyledonous and dicotyledonous differentiation. The phylogenetic analysis showed that Moso bamboo was closely related to monocotyledonous plants, such as rice, maize and *B. distachyon*, and the sequence similarity among members of the same subfamily is high, which may be derived from the evolution of the same gene, suggesting that its function may be conservative. Through analysis of the *PEPC* gene structure in bamboo, it was found that the PTPC and BTPC subfamily have significantly different structures. The PTPC subfamily has 9 introns and 10 exons, while the BTPC subfamily contains 20 exons and 19 introns [4]. Eight BTPC subfamily members (*PhePEPC3*, *DlaPEPC13*, *DlaPEPC17*, *DlaPEPC20*, *DhPEPC3*, *DhPEPC7*, *DhPEPC15*, *DhPEPC19*) contained 20 exons and 19 introns. The PTPC I subfamily (*OlaPEPC6*, *PhePEPC7*, *DlaPEPC6*, *DlaPEPC8*, *DhPEPC2*, *DhPEPC14*) only contained nine exons and eight introns, and *PhePEPC9* contained seven exons and seven introns; the remaining 47 *PEPC* genes all contained 10 exons and 9 introns, which is consistent with the gene structure of the PTPC subfamily. As is well known, the function of genes is closely related to their structure. An analysis of the *PhePEPC9* gene structure revealed that it is significantly different from other PTPC type members, indicating that it may have undergone functional differentiation during evolution. In addition, the conserved domain results of the bamboo PEPC protein showed that members of the family have highly conserved sequence characteristics, and all contain PEPcase domains, but there are slight differences in the location of the domains. PEPC contains multiple forms of isoenzymes. Multi-sequence alignment of the bamboo PEPC protein revealed that the bamboo PEPC proteins were C_3_ type, and there was no C_4_ type isoenzyme form. This is consistent with previous research, indicating that bamboo may be a species that primarily metabolizes energy through the C_3_ photosynthetic pathway [36]. A prediction of the conserved motifs in the PEPC proteins of the bamboo subfamily showed that except for PhePEPC9 lacking motifs 2 and 8, and OlaPEPC2 lacking motifs 3 and 6, all the other PTPC members contain 10 conserved motifs, and all the genes located in the same subfamily contain conserved domains with similar positions.

According to previous research, the expression of *PEPC* in specific organs and tissues may indicate its different biological functions. For example, *PEPC* plays different functions in Arabidopsis seedlings, castor and soybean roots and wheat leaves [50,51]. Interestingly, previous studies of the anatomical structure of the culm during the rapid growth period after bamboo shoots have found that chloroplasts in the culm of bamboo are mainly distributed in the basic tissues and cells around the vascular bundle sheath, which is similar to Kranz anatomy of C_4_ plants, indicating that the C_4_ photosynthetic pathway may exist in the culm of bamboo [34]. In this study, the expression levels of *PEPC* in different growth stages, flower organs and tissues of Moso bamboo showed that *PhePEPC1* is mainly specifically expressed in the culms of Moso bamboo, while *PhePEPC7*, *PhePEPC9*, and *PhePEPC10* are highly expressed in the culms and leaves of a non-flowering bamboo plant, indicating that these genes were likely involved in photosynthesis of bamboo culms. Furthermore, consistent with previous research, our findings further support the possible existence of a C_4_ photosynthetic pathway in the culm of bamboo. In addition, *PhePEPC4* and *PhePEPC5* were mainly expressed at high levels in winter bamboo shoots, suggesting that these genes may regulate material accumulation during the development of winter bamboo shoots. The expression patterns of *PhePEPC2*, *PhePEPC6* and *PhePEPC8* were similar, mainly playing a major regulatory role in the early stages of shoot growth and development. In addition, the expression patterns of *PhePEPC4* and *PhePEPC6* in the flower organs were similar. Except for a low expression in the leaves of a non-flowering bamboo plant and its glumes, they were highly expressed in immature embryos, flower buds and bracts and flagella, indicating that these two genes may regulate flower organ development, while *PhePEPC2* was specifically expressed in the flagella. The specific spatiotemporal expression pattern of its genes indicates that the functions of the *PEPC* gene family in Moso bamboo are complex and diverse. *PEPC* genes in non-photosynthetic organs, such as roots, also have a wide range of expression. *PhePEPC4*, *PhePEPC6*, *PhePEPC8* and *PhePEPC9* in the transcriptome data of Moso bamboo single-cell basal roots have confirmed this view. Root-type PEPC may be involved in plants’ response to stress, and it was suggested that root-type PEPC of bamboo may be involved in the regulation of stress response. Combining the tissue specificity and unique spatiotemporal expression pattern of different genes, the next research work can further focus on the regulation pattern of its genes, explore the mechanism of its differential expression and provide a new way to reveal the growth and development mechanism of Moso bamboo.

## 4. Materials and Methods

### 4.1. Identification and Characterization of PEPC in P. edulis, O. latifolia, D. latiflorus and D. brandisii

We downloaded the protein sequences of Arabidopsis *PEPC* family members from the *Arabidopsis thaliana* database TAIR (https://www.arabidopsis.org/, accessed on 21 April 2024) [52]. The genome sequences and annotation file information of *P. edulis*, diploid *O. latifolia* and hexaploid *D. latiflorus* and *D. brandisii* were obtained from the BambooBase (https://bamboo.genobank.org/, accessed on 11 May 2024) [53]. We downloaded the *PEPC* gene family (PF00311) hidden Markov models (HMM) online from the Pfam database (http://pfam.xfam.org/, accessed on 21 April 2024) [54]. We used the E value (E) < 10–40 as the threshold and HMMER 3.0 software to identify the *PEPC* genes in four bamboo species [55]. In addition, the conserved domain analysis in the NCBI database (https://www.ncbi.nlm.nih.gov/Structure/bwrpsb/bwrpsb.cgi, accessed on 11 May 2024) [56] was used to screen the retrieved candidate proteins and analyze whether these candidate protein sequences contained a complete PEPcase domain. Only the sequences with a complete PEPcase domain were considered to be *PEPC* family genes. We analyzed the physicochemical properties of the PEPC proteins of Bambusoideae through the online tool ExPASY (https://www.expasy.org/, accessed on 18 June 2024) [57].

To investigate the evolutionary relationships of the *PEPC* genes among different species, three algae (*Chlamydomonas reinhardtii V5.6*, *Micromonas pusilla V3.0* and *Ostreococcus lucimarinus V2.0*), *Physcomitrium patens V3.3*, *Selaginella moellendorffii V1.0*, six dicotyledonous plants’ (*Arabidopsis thaliana TAIR10*, *Gossypium raimondii V2.1*, *Populus trichocarpa V3.0*, *Solanum lycopersicum ITAG2.4*, *Glycine max Lee V1.1* and *Ricinus communis V0.1*) and six monocotyledonous plants’ (*Brachypodium distachyon V3.1*, *Oryza sativa V7.0*, *Zea mays RefGen_V4*, *Setaria italica V2.2*, *Sorghum bicolor V5.1* and *Saccharum officinarum V2.1*) PEPC family protein sequences were collected from the Phytozome V13.0 database (https://phytozome-next.jgi.doe.gov/, accessed on 21 April 2024) [58].

### 4.2. Evolutionary Analysis and Protein Multi-Sequence Alignment of PEPC in P. edulis, O. latifolia, D. latiflorus and D. brandisii

We used ClusterX2.1 software (V2.1.1) to align the PEPC protein sequences of 21 species, including *C. reinhardtii*, *O. lucimarinus*, *M. pusilla*, *P. patens*, *S. tamariscina*, *A. thaliana*, *G. max*, *R. communis*, *G. raimondii*, *P. trichocarpa*, *S. lycopersicum*, *O. sativa*, *Z. mays*, *B. distachyon*, *S. italica*, *S. bicolor*, *S. officinarum*, *P. edulis*, *O. latifolia*, *D. latiflorus* and *D. brandisii*, and used MEGA11 software (V11.0) to analyze the results [59]. We used the neighbor-joining method to set the self-expanding value to 1000 to build a rootless phylogenetic tree and visualized the results through the online website iTOL (http://itol.embl.de, accessed on 18 June 2024) [60].

### 4.3. Analysis of Gene Structure, Conservative Motif and Promoter Cis-Regulatory Element

GSDS2.0 (http://gsds.gao-lab.org/, accessed on 18 June 2024) software was used to analyze the gene structure of the introns, exons and other genes of each member of the bamboo *PEPC* family, and TBtools (V2.096) was used for visual mapping [61,62]. We used the online tool MEME Version 4.12.0 (http://memesuite.org/tools/meme, accessed on 18 June 2024) [63] to analyze the protein conserved motifs of bamboo *PEPC* family members, and set the motif retrieval value to 10. According to the whole genome data of *P. edulis*, the promoter subsequence encoding the *PEPC* gene of Moso bamboo (2000 bp upstream of the transcription start site) was extracted, and we predicted the cis acting elements through PlantCARE (http://bioinformatics.psb.ugent.be/webtools/plantcare/html/, accessed on 18 June 2024) [64]. Finally, we visualized the results using TBtools [62].

### 4.4. Collinearity Analysis and the Ka/Ks Ratio of P. edulis, O. latifolia, D. latiflorus and D. brandisii PEPC Genes

We performed a collinearity analysis of four bamboo *PEPC* families using the method described by previous research [65]. According to previous research methods, *Ka*/*Ks* values between *PEPC* homologous gene pairs were calculated using TBtools for selection pressure analysis [66].

### 4.5. Analysis of Gene Expression Patterns

In order to study the expression levels of the *PEPC* genes in different tissues and shoots at different developmental stages, the transcriptome data measured were from previous studies [39]. These included different developmental stages of Moso bamboo shoots and different organs, as well as single-cell transcriptome data of Moso bamboo basal root [40]. We measured the relative expression abundance of different tissues based on previous research [39], and the hotspot map was drawn by TBtools software (V2.096).

### 4.6. RNA Extraction, cDNA Synthesis and qRT-PCR

The total RNA was extracted according to a Prime ScriptTM RT reagent Kit. RNA reverse transcription was performed with a gDNA Eraser (TaKaRa) kit. According to the SYBR Green I Master (Roche) kit protocol, the qRT-PCR system was set up: SYBR Master Mix 10 μL, upstream primer 0.4 μL, downstream primer 0.4 μL, template cDNA 2 μL and added ddH_2_O 20 μL. Using Roche LightCycler480^®^ System, the sample was amplified by qRT-PCR with *PheTIP41* as the internal reference [67]. Three biological replicates were performed to calculate the relative expression of the target gene. The list of primers is shown in Table S3.

### 4.7. Subcellular Localization Experiment of PhePEPC Proteins in Moso Bamboo

The subcellular localization fusion vector was constructed by seamless cloning. The PCR product of *PhePEPC6* was digested with the restriction endonuclease KpnI in a linear plant binary vector pCAMBIA2300-35S-eGFP connection. Enzyme digestion of the carrier was performed according to the instructions of endonuclease KpnI (1068S) cutting system (20 μL): endonuclease 1 μL, endonuclease buffer 1 μL, plasmid DNA 1 μg, ddH_2_O supplementation to 20 μL. Purification was performed after 37 °C 2 h. Subcellular localization of the PhePEPC6 protein was analyzed with reference to the transient transformation method of tobacco leaves [68]. After injecting the infective solution through the back of the leaf, we cultured it in low light or dark light for 3–5 days, then performed eGFP by confocal laser microscopy observation of fluorescence.

### 4.8. Plant Material, Growth Conditions and Treatments

Moso bamboo seedlings and Bentley tobacco seedings were germinated in long daylight incubators maintained at 23 °C and 24 °C, respectively. The light intensity was 140 μmol photons m^−2^s^−1^, and the relative humidity was 70%. Three-month-old Moso bamboo seedlings of the same size were irrigated with 100 μM GA_3_. After treatment for 0, 3, 6, 12 and 24 h, the bamboo roots, culms, and leaves were collected as experimental materials and quickly frozen in liquid nitrogen before being stored at −80 °C. Moso bamboo seedlings irrigated with water were used as the control, and biological repeats were performed 3 times for each treatment. In order to study the subcellular localization of PEPC protein in bamboo, 4-week-old tobacco leaves were selected for a subcellular localization experiment, and the third to fifth leaves were selected for agrobacterium injection. The experiment was repeated three times.

### 4.9. Statistical Analysis

The statistical analysis was conducted using SPSS software (V27.0), with three biological replicates set for all the experiments. We used Student’s testing to assess the differences (0.01 ≤ *p* ≤ 0.05 *, *p* < 0.01 **).

## 5. Conclusions

This study identified the members of the *PEPC* gene family from diploid bamboo to hexaploid bamboo and conducted a systematic bioinformatics analysis of the *PEPC* gene family in the Bambusoideae. The investigation uncovered stark disparities in the abundance of *PEPC* gene members across this polyploidy transition, hinting at a potential role of chromosome duplication in the amplification of *PEPC* genes. The collinearity analysis suggests that the amplification of *PEPC* genes from diploid to hexaploid may be due to chromosome duplication. Further analysis of the gene structure and phylogenetic status of *PEPC* members revealed that the majority of *PEPC* members are relatively conserved in Bambusoideae, and *PhePEPC9* may emerge as a potential outlier exhibiting functional divergence due to its distinct structural differences. In addition, through promoter analysis and expression pattern analysis, it was found that the *PEPC* gene can respond to hormone signals and abiotic stress, such as drought and low temperature. It is noteworthy that *PhePEPC6*, *PhePEPC7* and *PhePEPC9* are highly expressed in leaves under exogenous GA_3_ application, suggesting that these genes may be involved in leaf growth and development under the GA pathway. Moreover, subcellular localization experiments confirmed that PhePEPC6 protein plays a critical role on the cell membrane. In summary, this study screened and identified *PEPC* genes in Bambusoideae and found that they respond to exogenous GA signals, providing a reference for delving deeper into the mechanism by which the *PEPC* gene regulates photosynthesis in bamboo (Table S1).

## Data Availability

All the data to support the study results in this paper are in the Supplementary Materials.

## Supplementary Materials

The following supporting information can be downloaded at: https://www.mdpi.com/article/10.3390/plants13172426/s1, Figure S1: Multiple sequence alignment of PEPC proteins in *P. edulis*, *D. latiflorus* and *Z. mays*; Figure S2: Multiple sequence alignment of PEPC proteins in *P. edulis* and *D. brandisii*; Figure S3: Expression patterns of *PhePEPCs* in single-cell transcriptome cell clusters of Moso bamboo basal roots; Figure S4: RNA nucleic acid gel electrophoresis of different tissues of Moso bamboo seedlings treated with GA; Figure S5: Numerical analysis of RNA integrity in different tissues of Moso bamboo seedlings treated with GA; Figure S6: Melting curves analysis of *PhePEPC6*, *PhePEPC7*, *PhePEPC9* in RT-PCR experiments after GA treatment; Table S1: Physicochemical properties of proteins of *PEPC* gene family members of *P. edulis*, *O. latifolia*, *D. latiflorus* and *D. brandisii*; Table S2: The *Ka*/*Ks* values of homologous *PEPC* pairs in *P. edulis*, *O. latifolia* and *D. latiflorus*; Table S3: The primer sequences used in this study.
