# Supplementary material for: Conservation and Divergence of PEPC Gene Family in Different Ploidy Bamboos
Source: Plants (Basel). 2024 Aug 30;13(17):2426. doi: 10.3390/plants13172426 (PMC11397392; doi:10.3390/plants13172426)
Supplement: Supplementary file 1 [file plants-13-02426-s001.zip › Figure S1. Multiple sequence alignment of PEPC proteins in P. edulis, D. latiflorus and Z. mays.pdf]

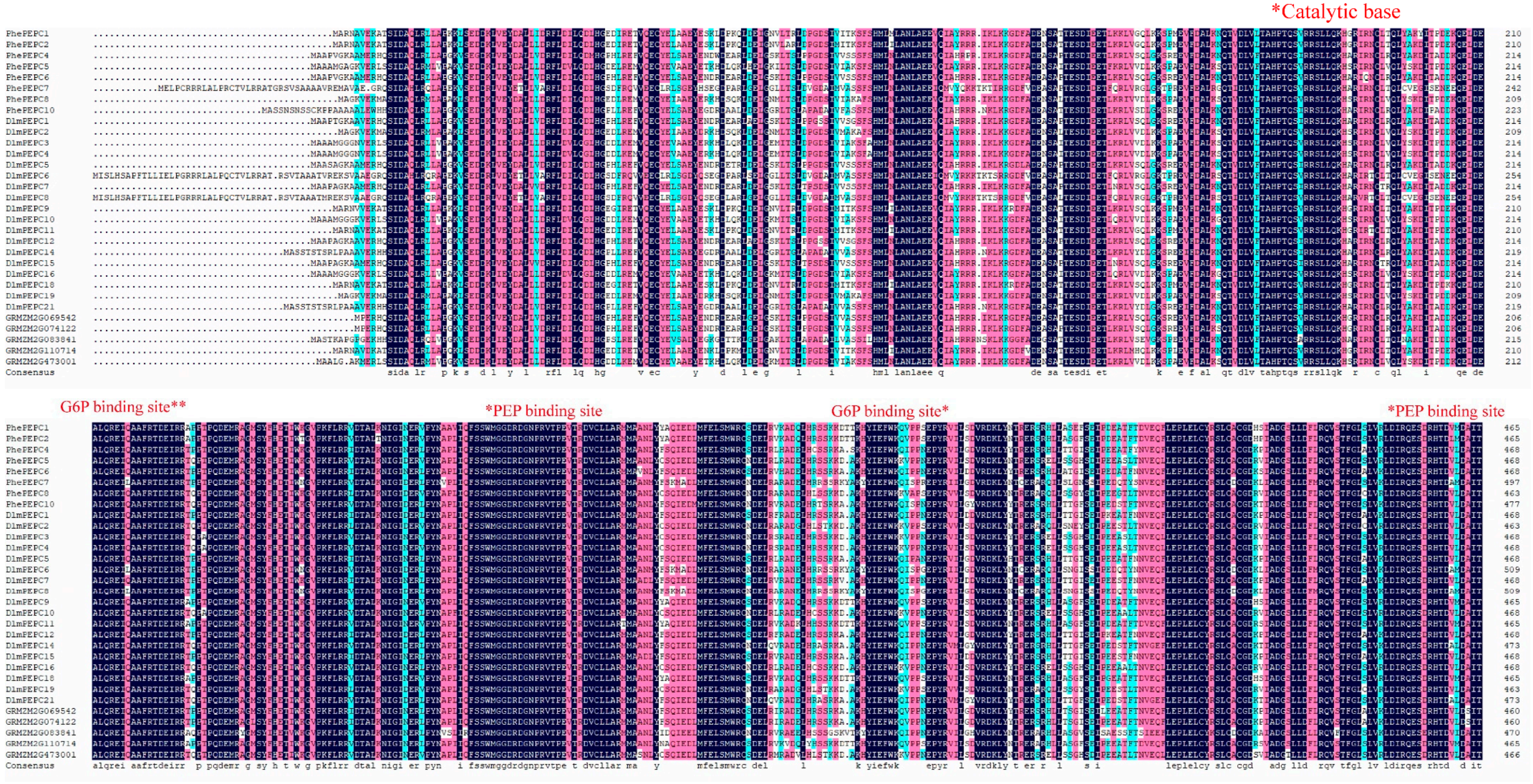
*Catalytic base
G6P binding site**
*PEP binding site
G6P binding site*
*PEP binding site

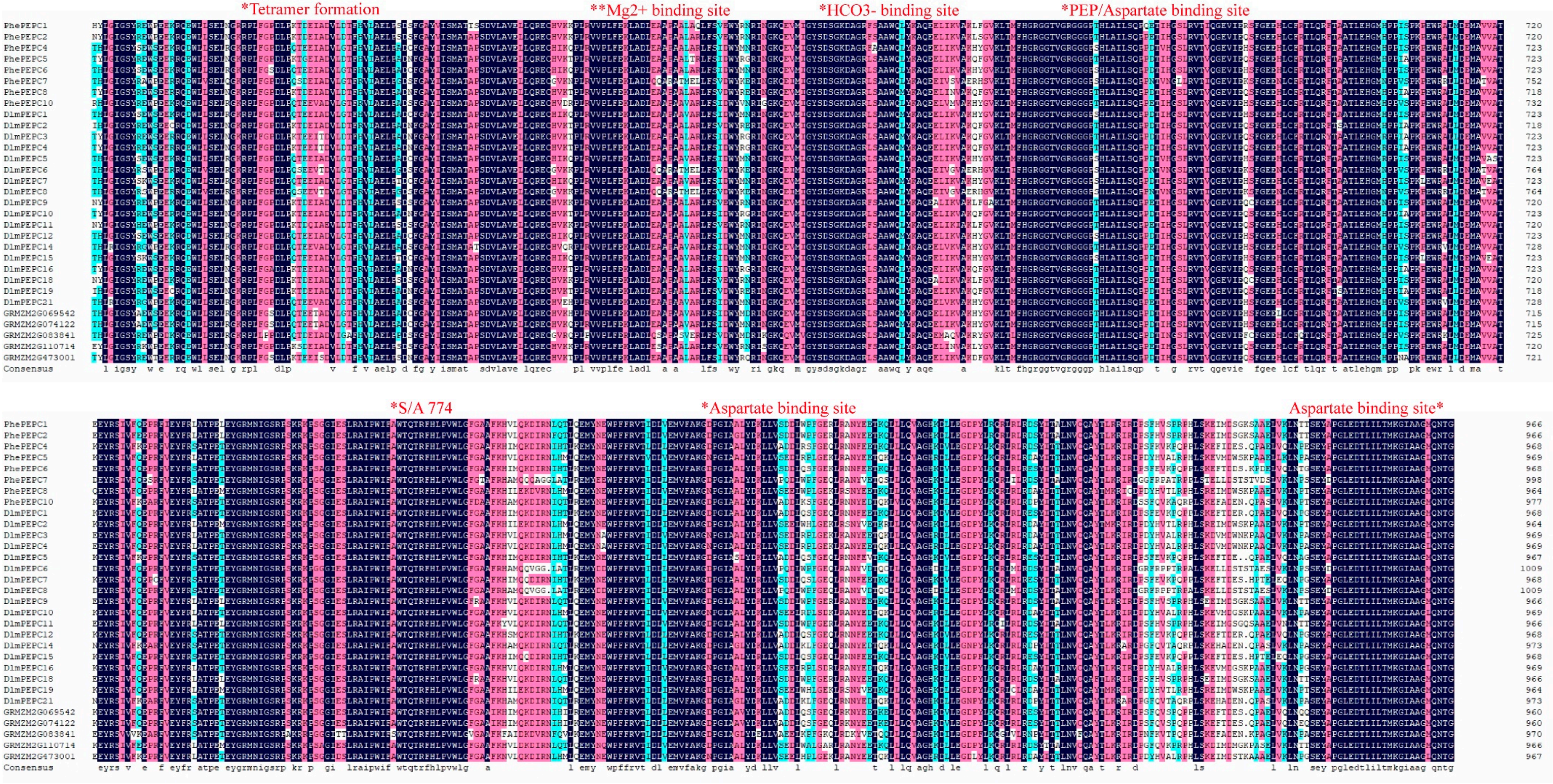


**Figure S1.** Multiple sequence alignment of PEPC proteins in *P. edulis*, *D. latiflorus* and *Z. mays*. The red asterisk represents the conserved functional sites of the PEPC protein family.
