# Supplementary material for: Conservation and Divergence of PEPC Gene Family in Different Ploidy Bamboos
Source: Plants (Basel). 2024 Aug 30;13(17):2426. doi: 10.3390/plants13172426 (PMC11397392; doi:10.3390/plants13172426)
Supplement: Supplementary file 1 [file plants-13-02426-s001.zip › Figure S2. Multiple sequence alignment of PEPC proteins in P. edulis and D. brandisii.pdf]

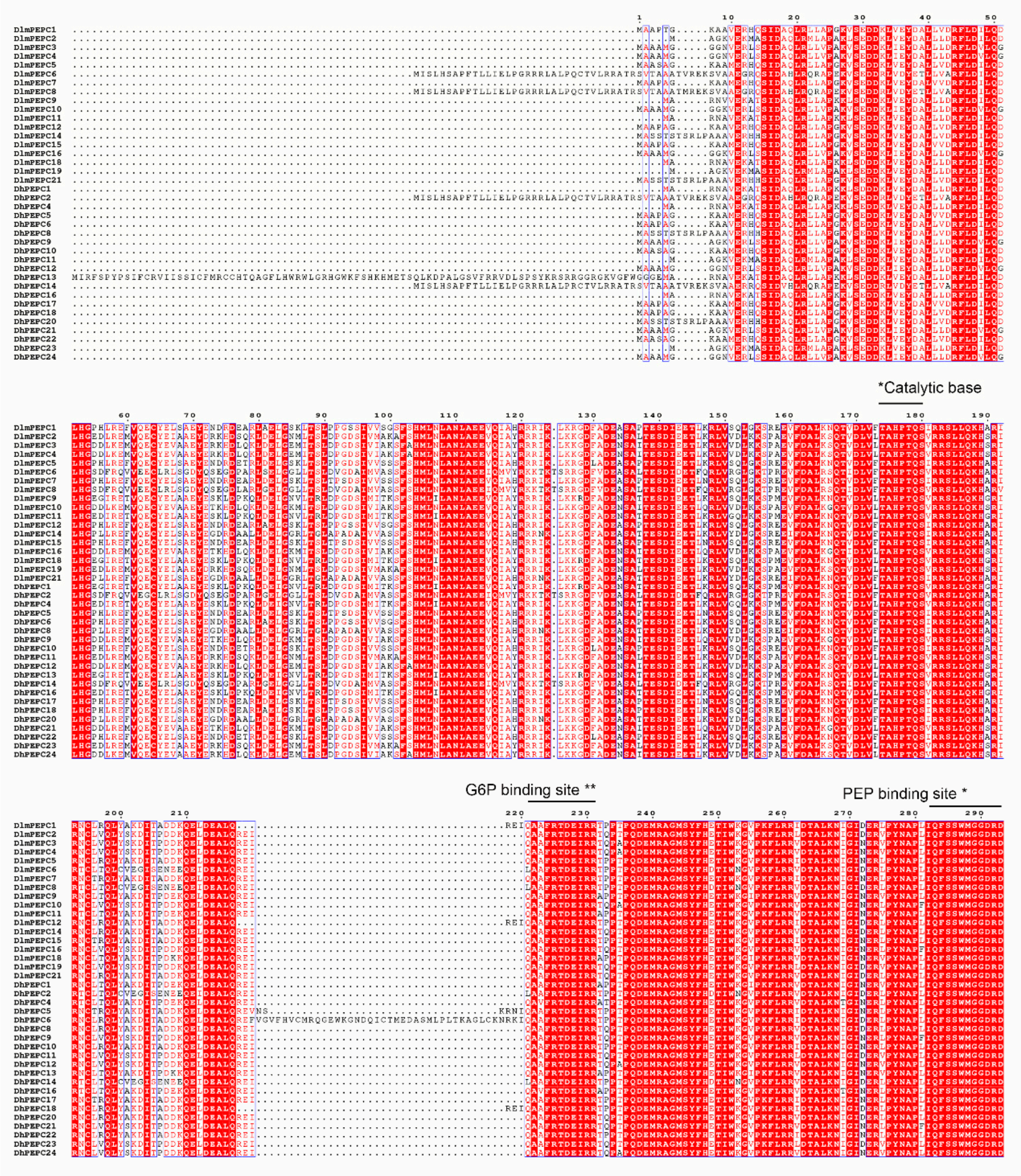
*Catalytic base
G6P binding site **
PEP binding site *

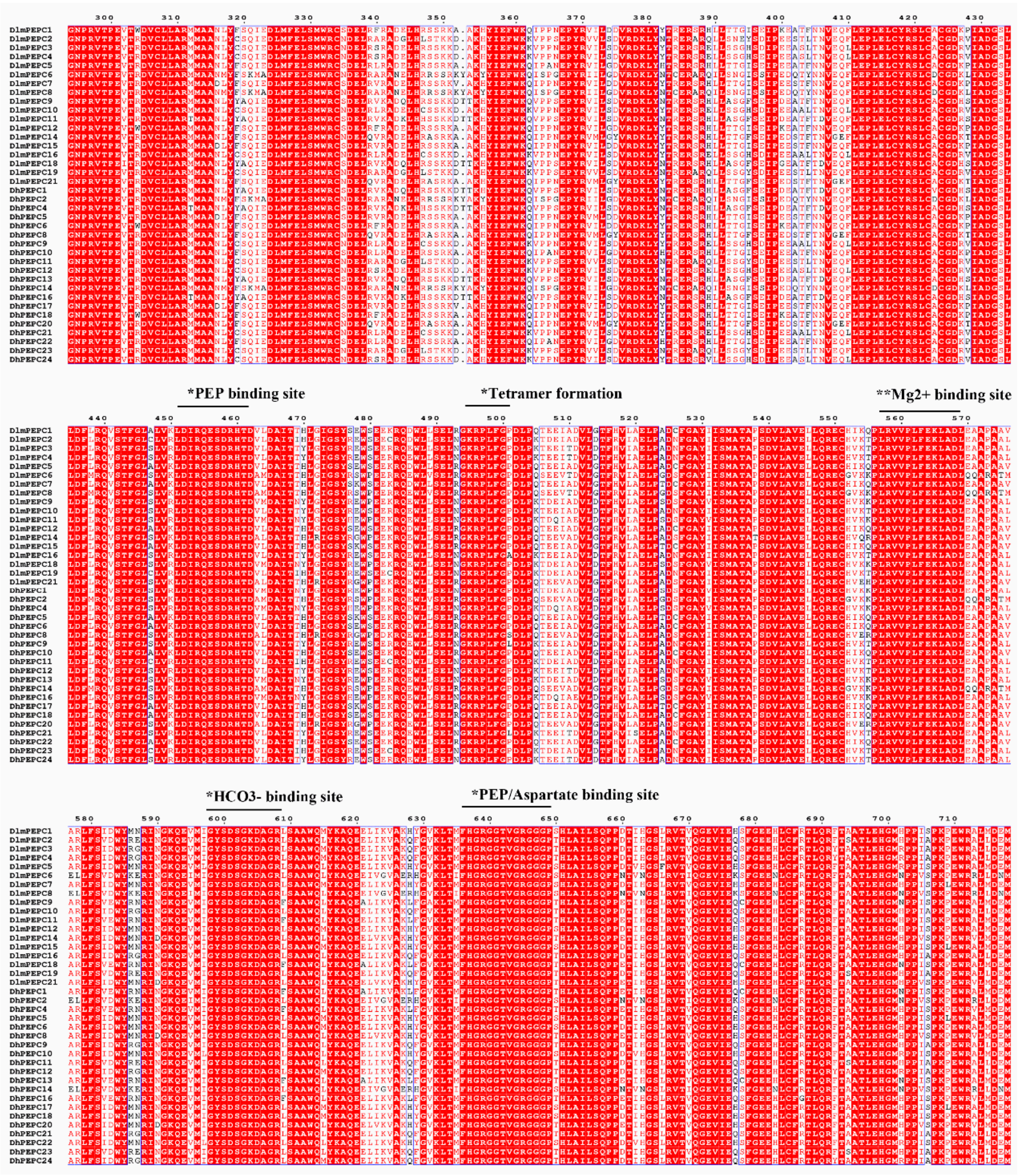
*PEP binding site
*Tetramer formation
**Mg2+ binding site
*HCO3- binding site
*PEP/Aspartate binding site

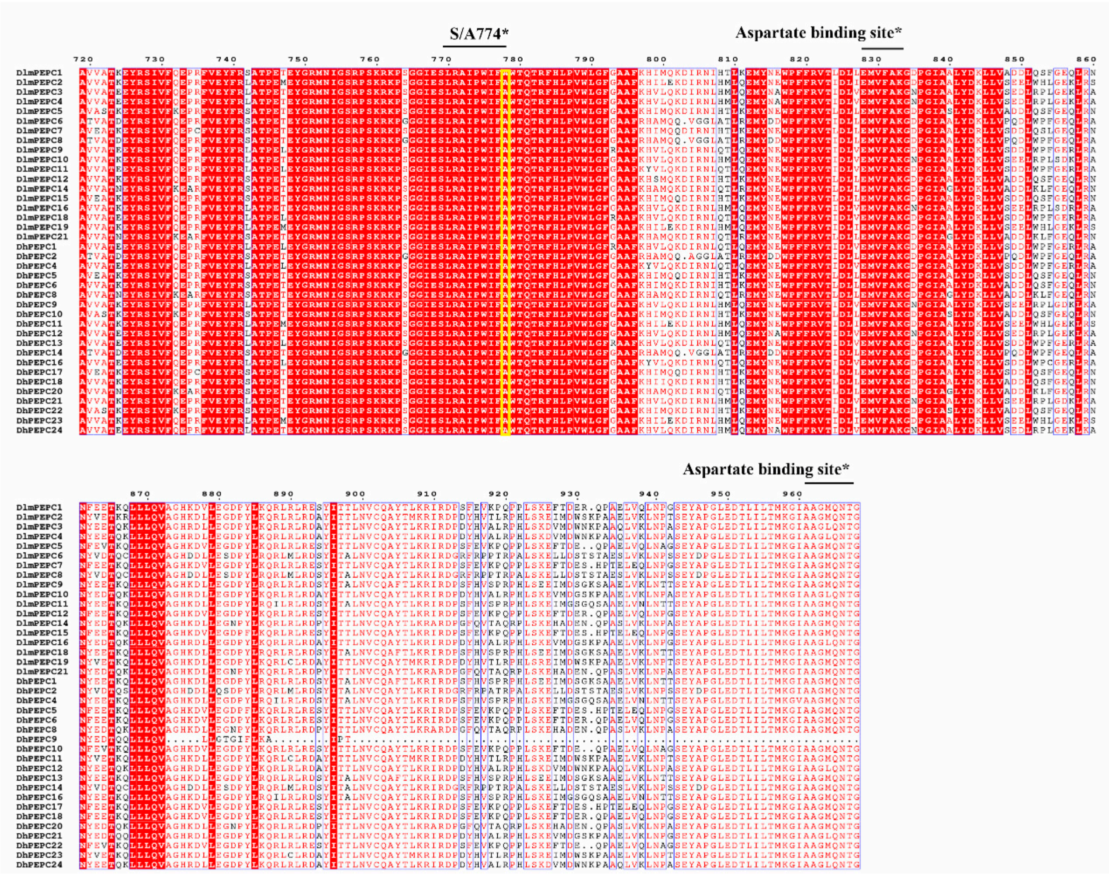

Figure S2. Multiple sequence alignment of PEPC proteins in *P. edulis* and *D. brandisii*. The dash represents the conserved sites of the PEPC family, and the yellow box represents 774 amino acid residues. * / ** represents conserved amino acids with proven function.
