# Supplementary material for: Conservation and Divergence of PEPC Gene Family in Different Ploidy Bamboos
Source: Plants (Basel). 2024 Aug 30;13(17):2426. doi: 10.3390/plants13172426 (PMC11397392; doi:10.3390/plants13172426)
Supplement: Supplementary file 1 [file plants-13-02426-s001.zip › Figure S3. Expression patterns of PhePEPCs in single cell transcriptome cell clusters of Moso bamboo basal roots.pdf]

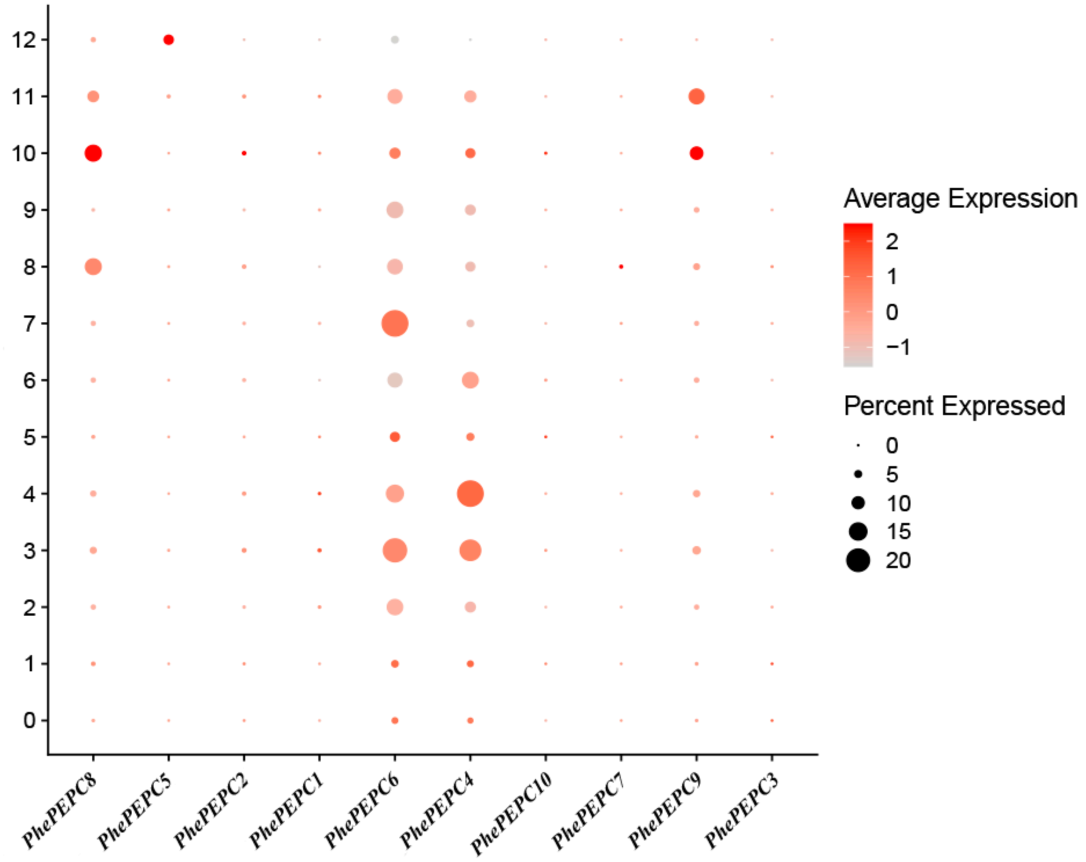

Figure S3. Expression patterns of *PhePEPCs* in single cell transcriptome cell clusters of Moso bamboo basal roots. Clusters 0, 1, 5 and 10 represent ground tissues; Cluster 2 represents the transition cell; Clusters 3 and 4 represent undefined tissues; Cluster 6 represents the epidermis; Clusters 7, 8, 11 and 12 represent the root cap; Cluster 9 represents the initial cell. The dot diameter shows the proportion of cluster cells expressing a given gene; the color shows expression across cells in the cluster.
