# Supplementary material for: Conservation and Divergence of PEPC Gene Family in Different Ploidy Bamboos
Source: Plants (Basel). 2024 Aug 30;13(17):2426. doi: 10.3390/plants13172426 (PMC11397392; doi:10.3390/plants13172426)
Supplement: Supplementary file 1 [file plants-13-02426-s001.zip › Figure S4. RNA nucleic acid gel electrophoresis of different tissues of Moso bamboo seedlings treated with GA.pdf]

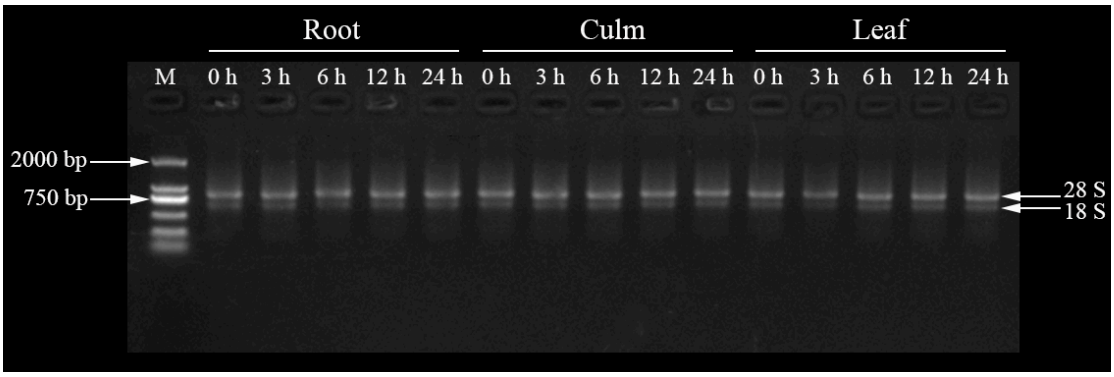

Figure S4. RNA nucleic acid gel electrophoresis of different tissues of Moso bamboo seedlings treated with GA. M: Maker with a molecular weight of 2000 bp.
