# Supplementary material for: Conservation and Divergence of PEPC Gene Family in Different Ploidy Bamboos
Source: Plants (Basel). 2024 Aug 30;13(17):2426. doi: 10.3390/plants13172426 (PMC11397392; doi:10.3390/plants13172426)
Supplement: Supplementary file 1 [file plants-13-02426-s001.zip › Figure S5. Numerical analysis of RNA integrity in different tissues of Moso bamboo seedlings treated with GA.pdf]

Root-0h

A

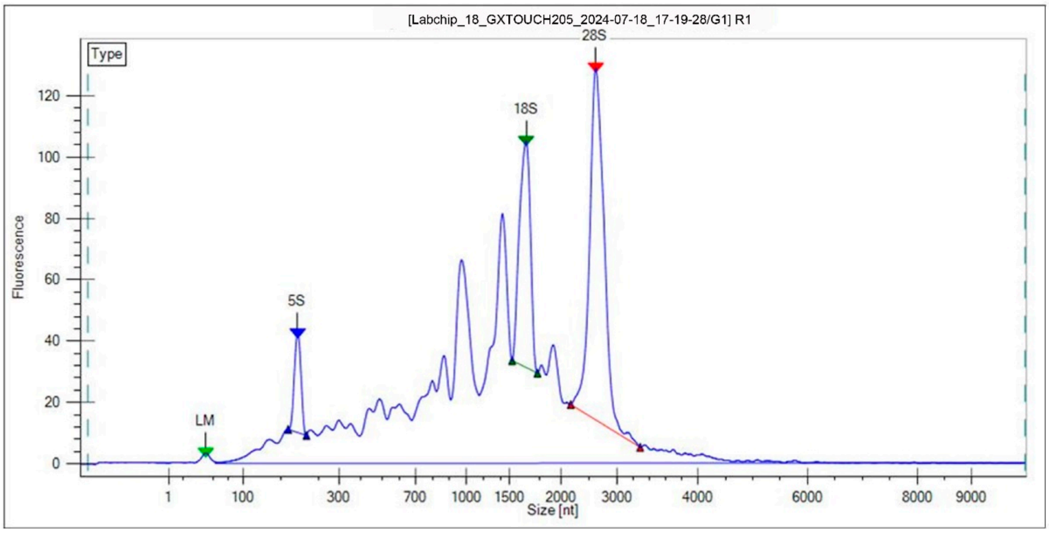

Root-3h

B

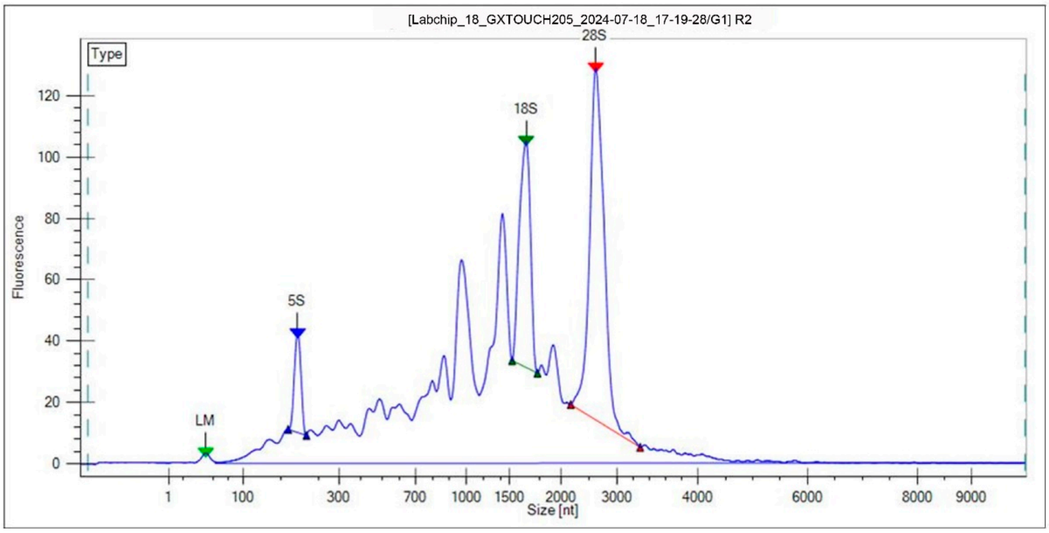

Root-6h

C

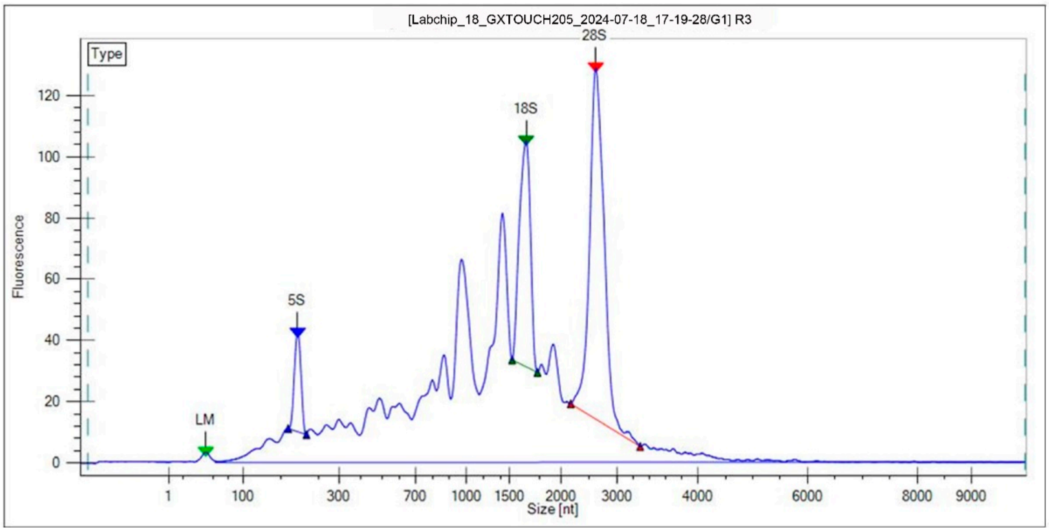

Root-12h

D

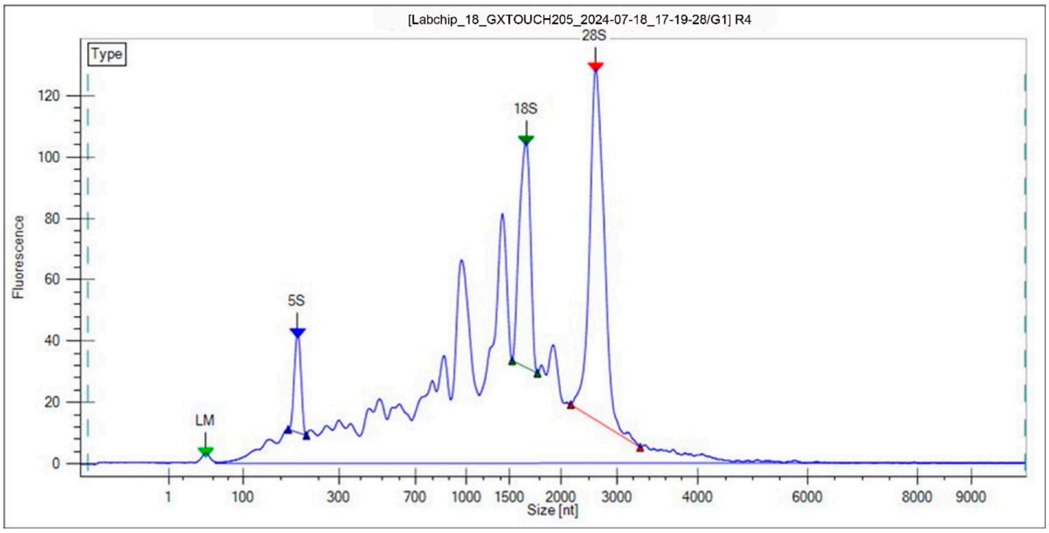

Root-24h

E

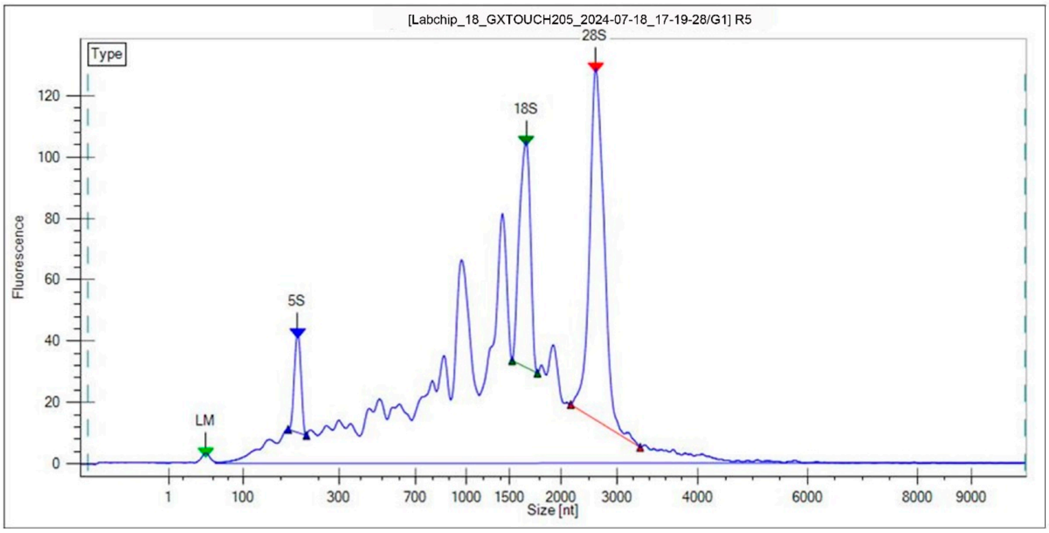

Culm-0h

F

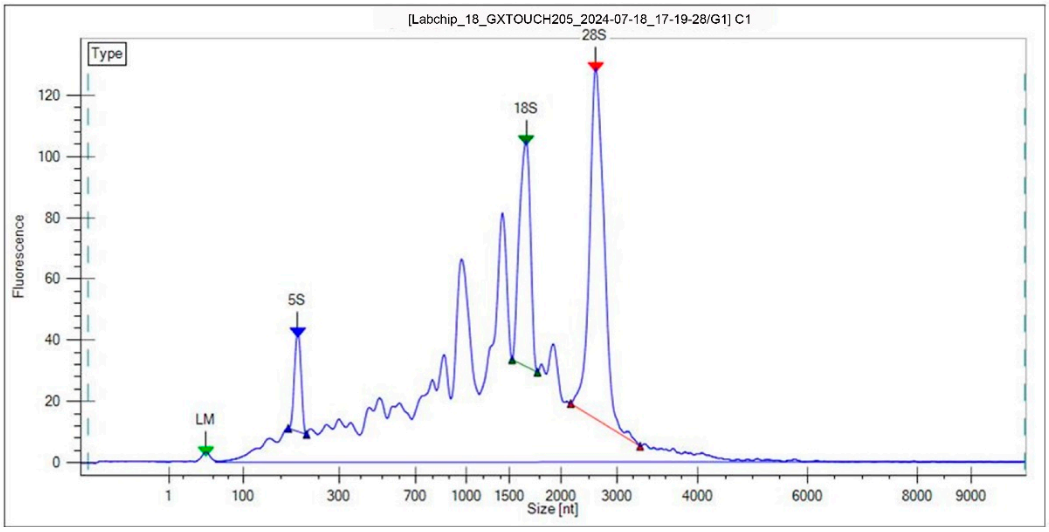

Culm-3h

G

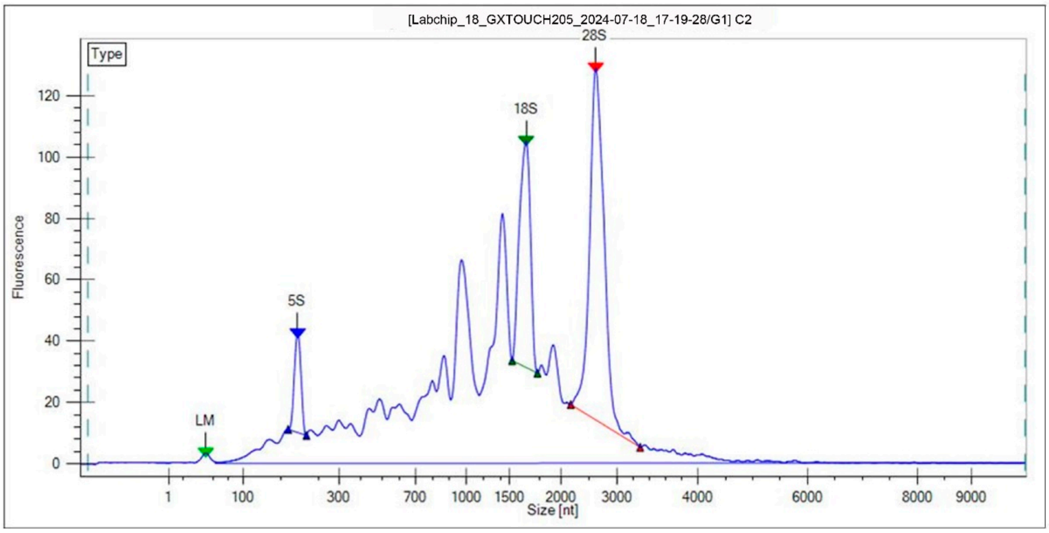

Culm-6h

H

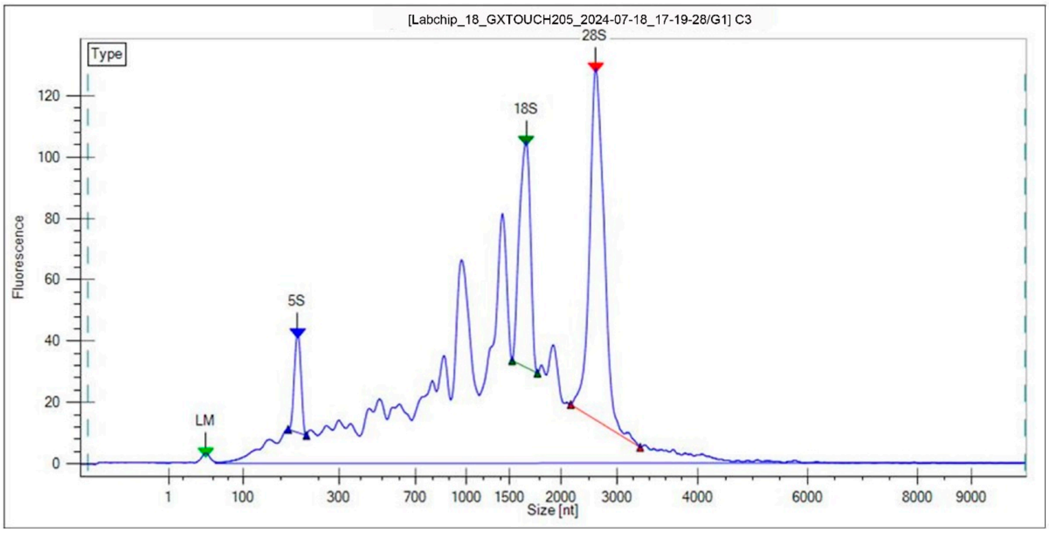

Culm-12h

I

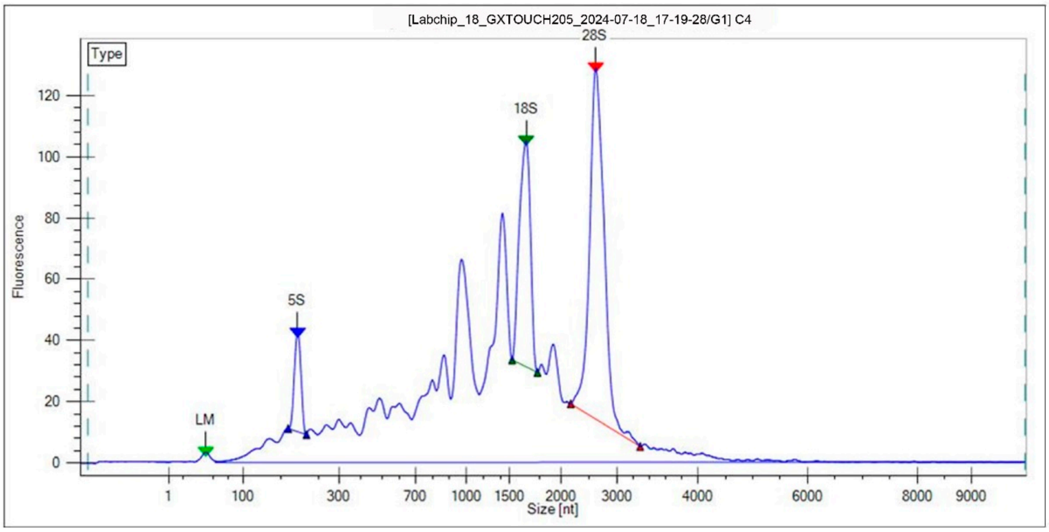

Culm-24h

J

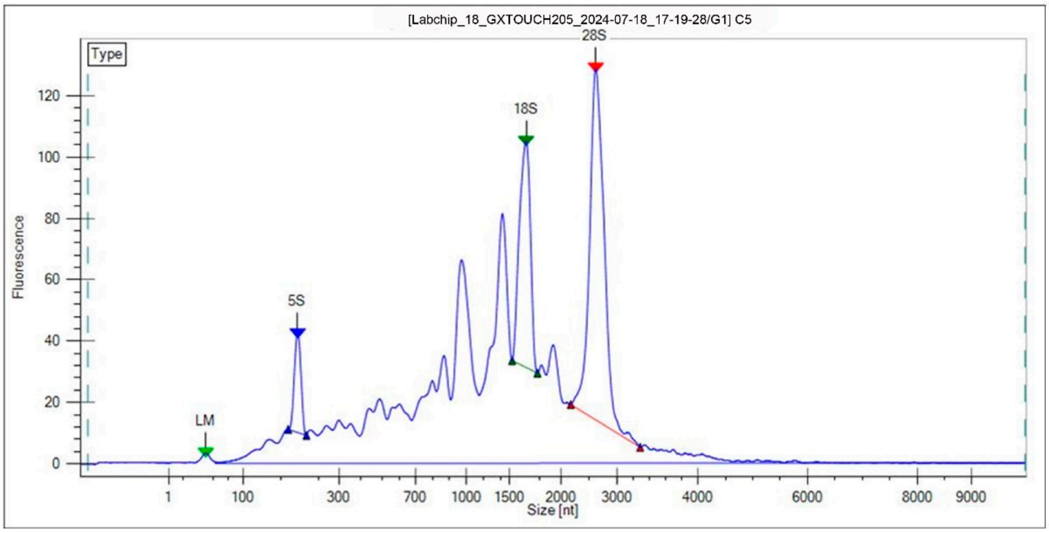

Leaf-0h

K

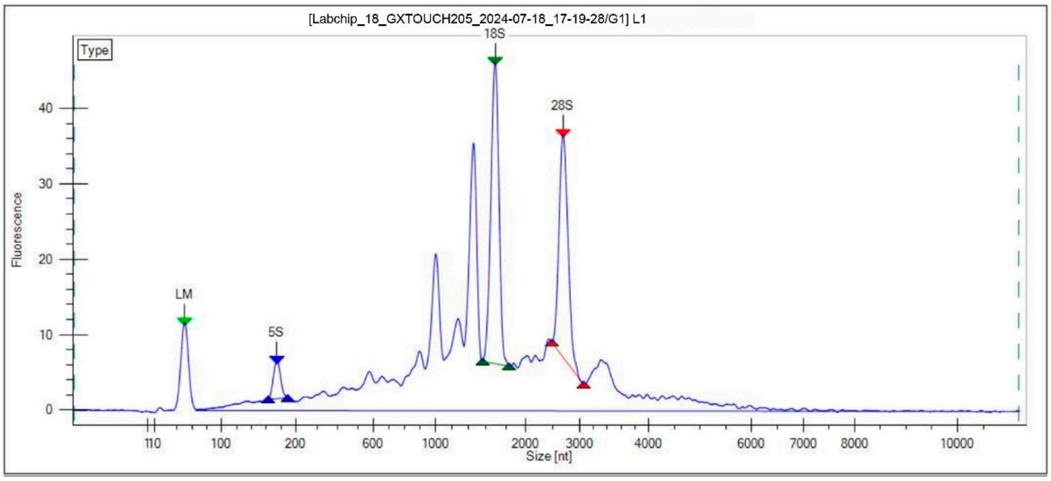

Leaf-3h

L

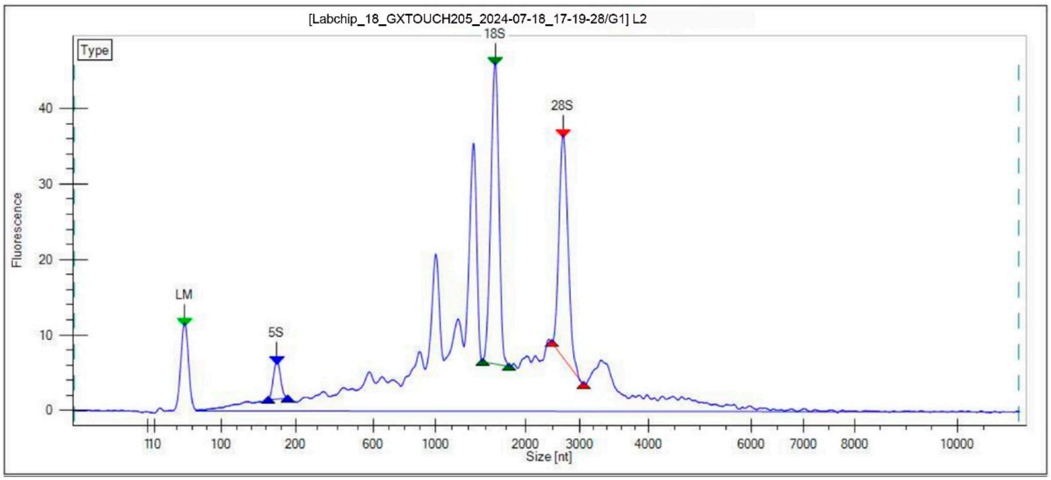

M

Leaf-6h

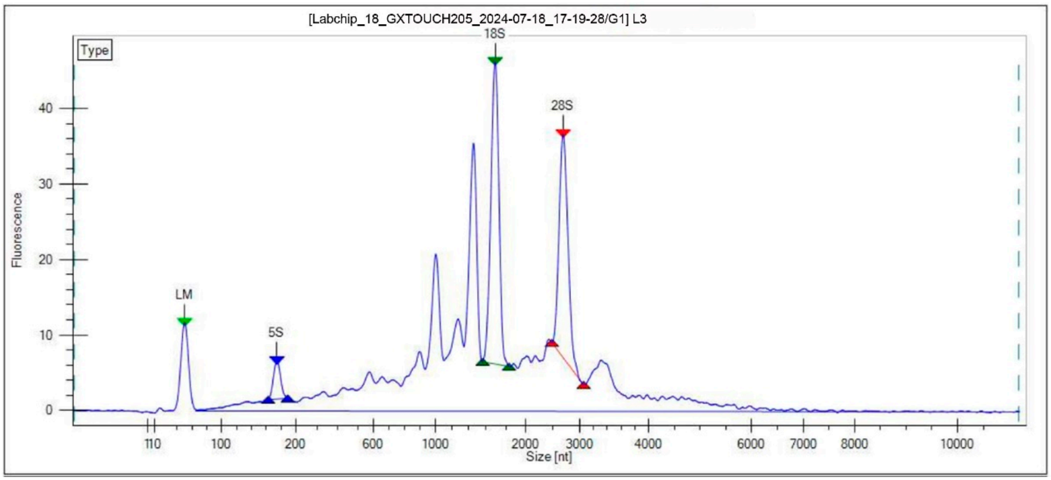

N

Leaf-12h

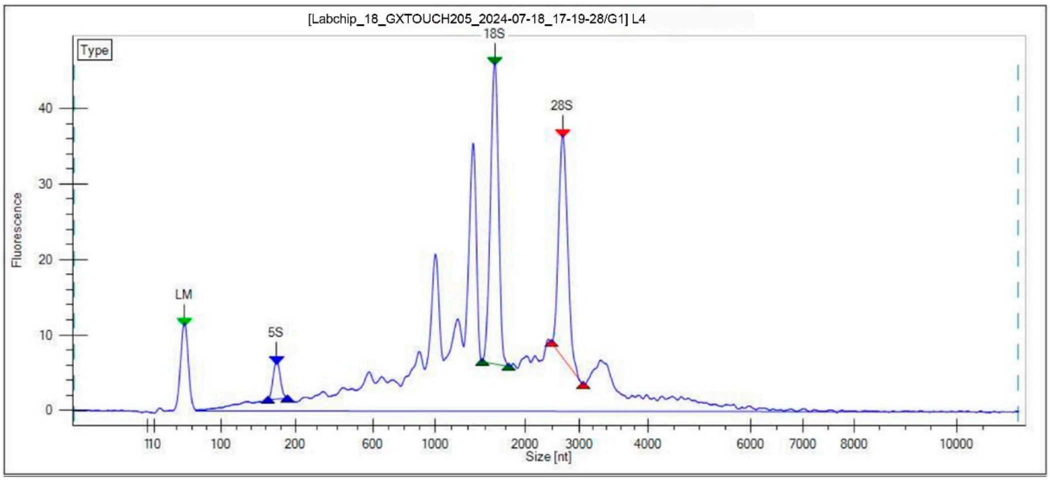

O

Leaf-24h

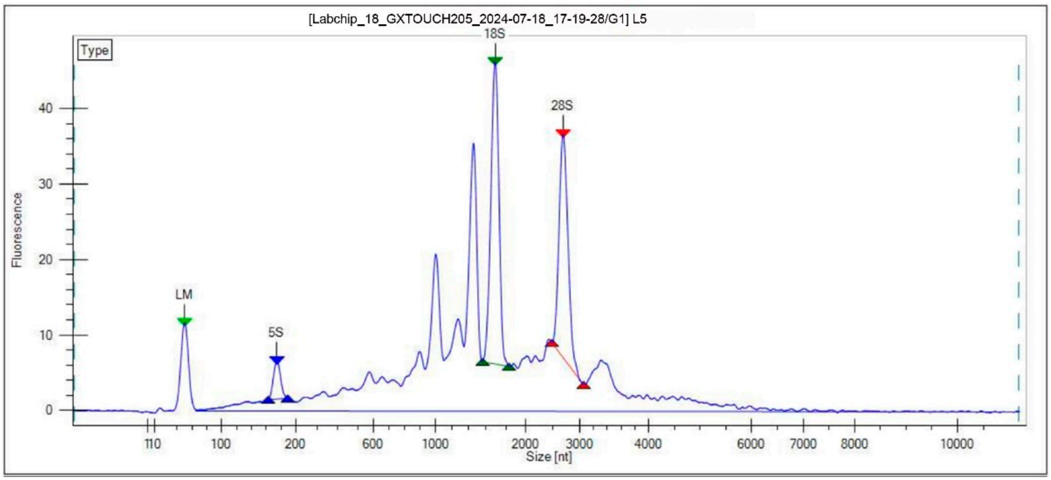

Figure S5. Numerical analysis of RNA integrity in different tissues of Moso bamboo seedlings treated with GA. A-E: Root tissues RIN (RNA integrity number) at different time periods after GA processing. F-J: Culm tissues RIN at different time periods after GA processing. K-O: Leaf tissues RIN at different time periods after GA processing.
