# Supplementary material for: Conservation and Divergence of PEPC Gene Family in Different Ploidy Bamboos
Source: Plants (Basel). 2024 Aug 30;13(17):2426. doi: 10.3390/plants13172426 (PMC11397392; doi:10.3390/plants13172426)
Supplement: Supplementary file 1 [file plants-13-02426-s001.zip › Figure S6. Melting curves analysis of PhePEPC6, PhePEPC7, PhePEPC9 in RT-PCR experiments after GA treatment.pdf]

Root-*PhePEPC6*

A

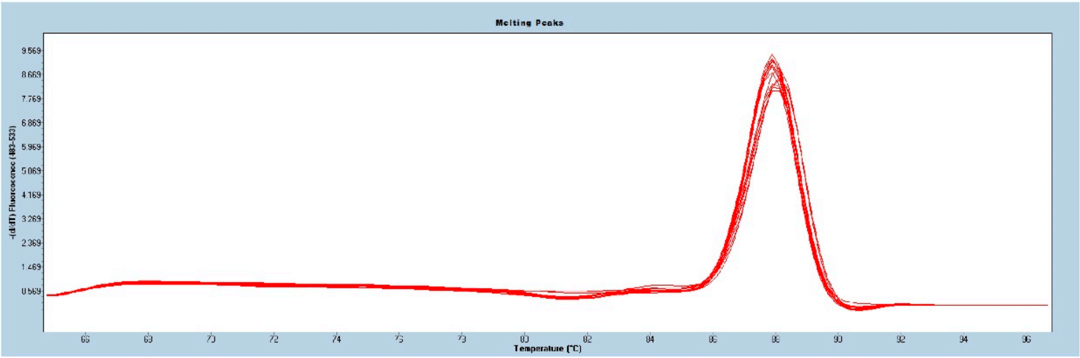

Root-*PhePEPC7*

B

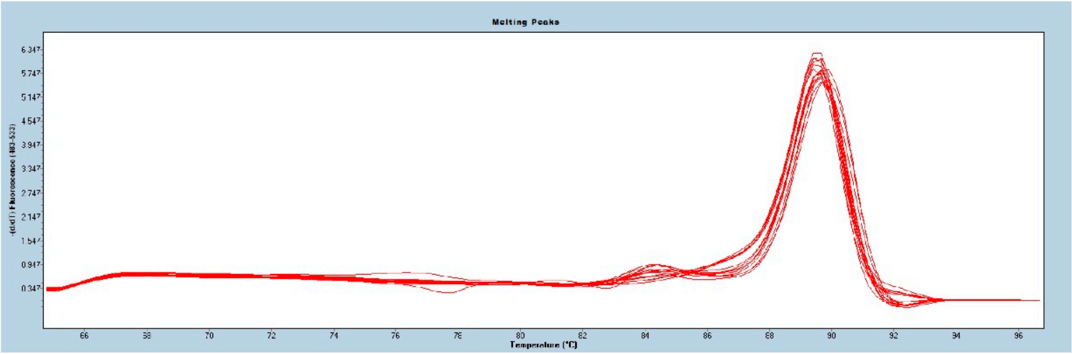

Root-*PhePEPC9*

C

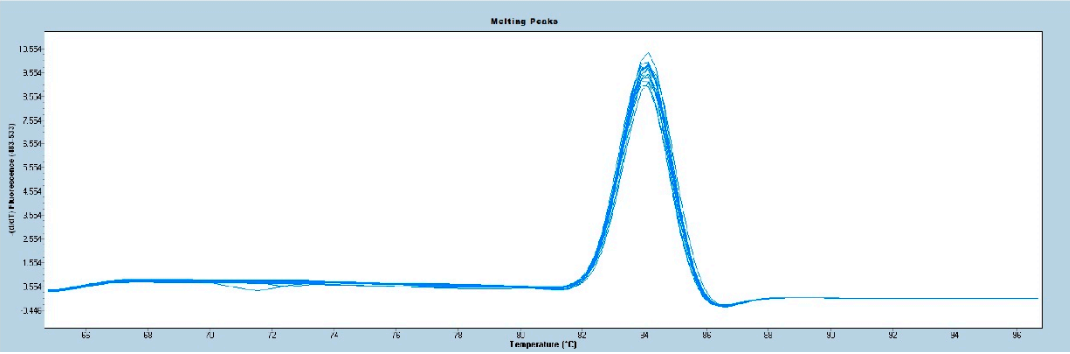

Root-*PheTIP41*

D

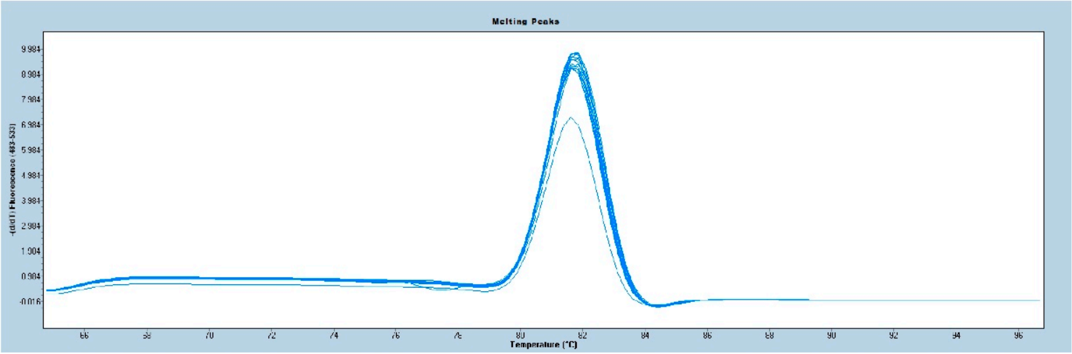

Culm-*PhePEPC6*

E

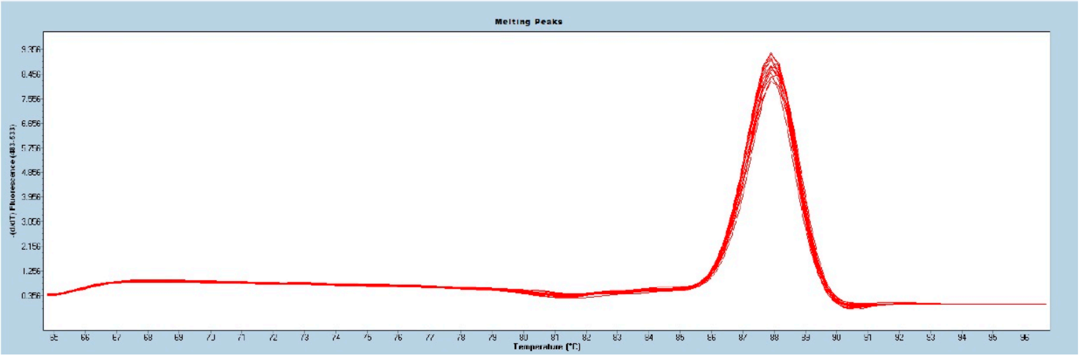

Culm-*PhePEPC7*

F

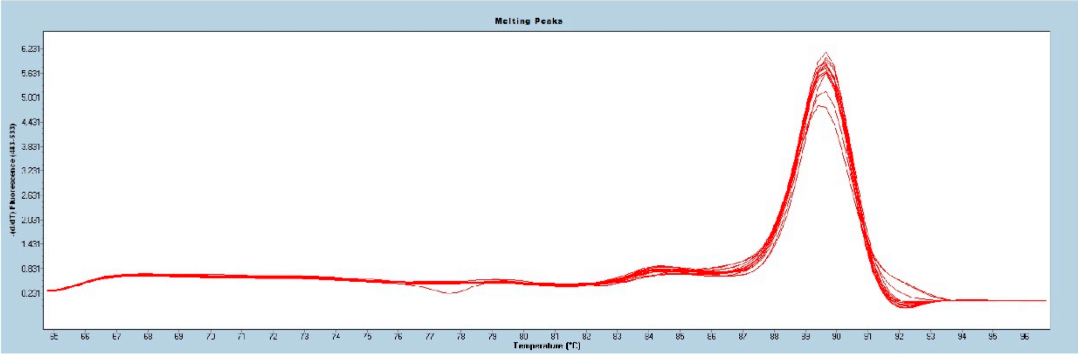

Culm-*PhePEPC9*

G

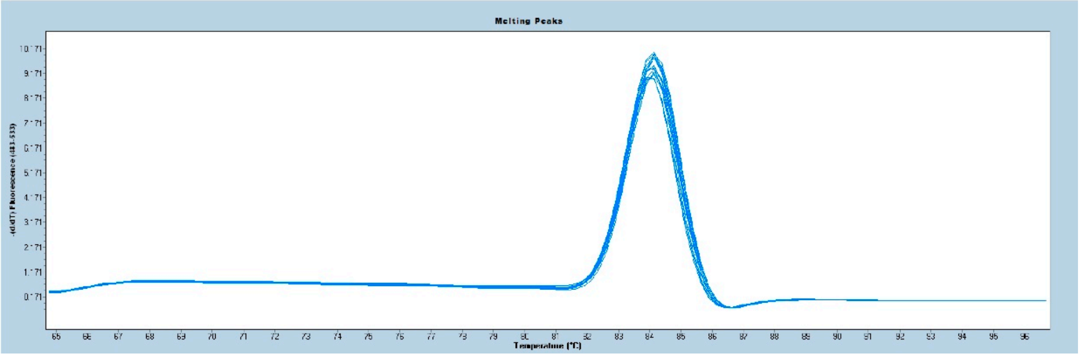

Culm-*PheTIP41*

H

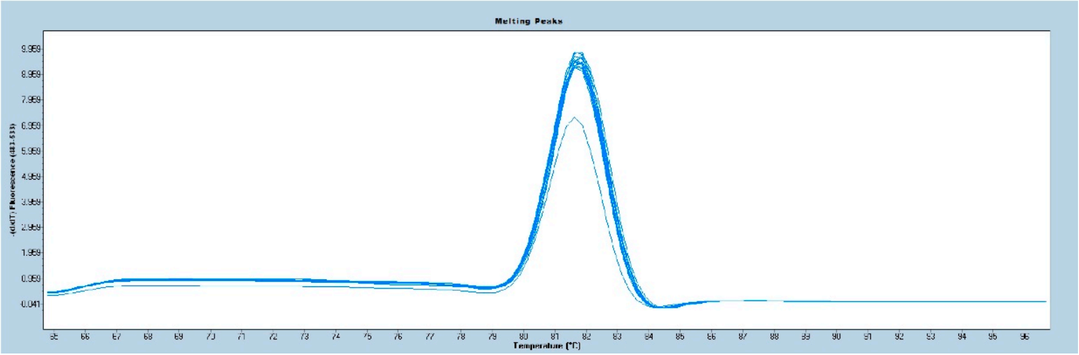

I

Leaf-*PhePEPC6*

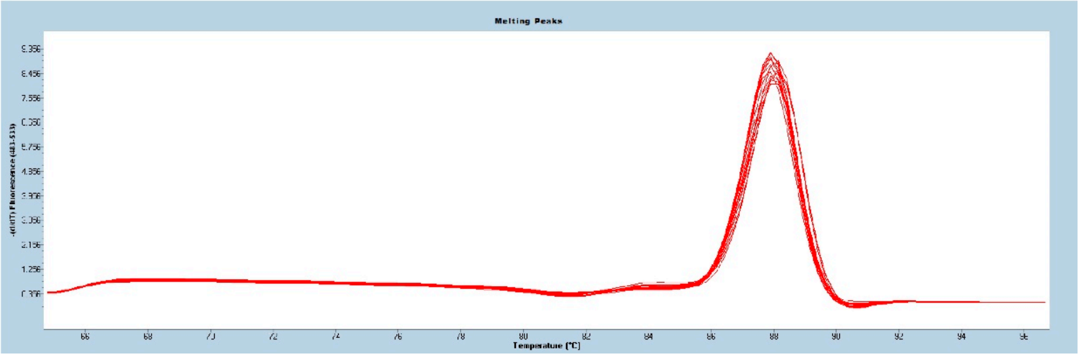

J

Leaf-*PhePEPC7*

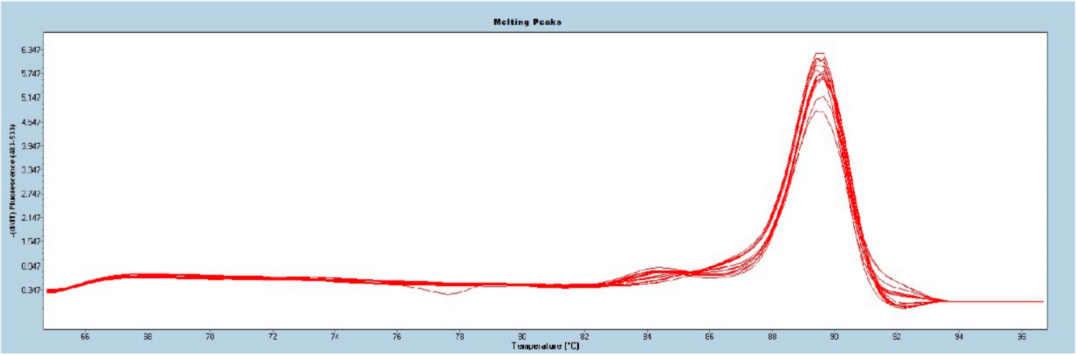

K

Leaf-*PhePEPC9*

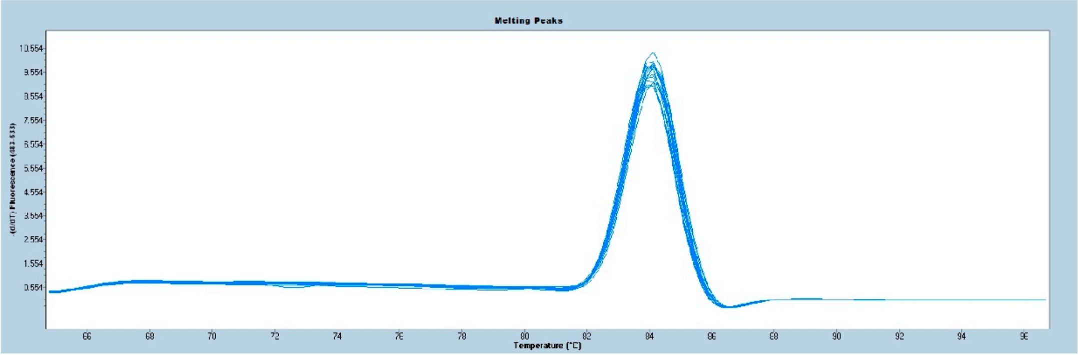

L

Leaf-*PheTIP41*

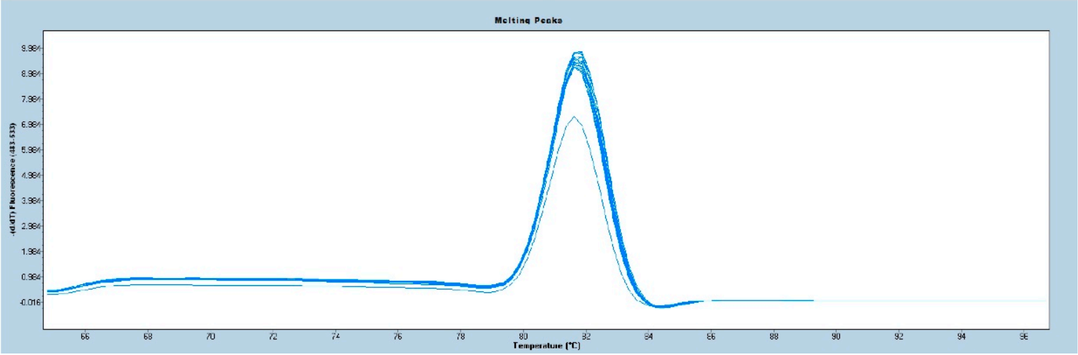

Figure S6. Melting curves analysis of *PhePEPC6*, *PhePEPC7*, *PhePEPC9* in RT-PCR experiments after GA treatment. The horizontal axis represents temperature, and the vertical axis represents the change in fluorescence signal per unit time. *PheTIP41* as reference gene in Moso bamboo.
